# The urinary microbiome shows different bacterial genera in renal transplant recipients and non-transplant patients at time of acute kidney injury – a pilot study

**DOI:** 10.1186/s12882-020-01773-1

**Published:** 2020-04-06

**Authors:** Daniela Gerges-Knafl, Pichler Peter, Alexander Zimprich, Christoph Hotzy, Wolfgang Barousch, Rita M. Lang, Elisabeth Lobmeyr, Sabina Baumgartner-Parzer, Ludwig Wagner, Wolfgang Winnicki

**Affiliations:** 1 Department of Internal Medicine III, Division of Nephrology and Dialysis, Medical University of Vienna, Waehringer Guertel 18-20, 1090 Vienna, Austria; 2 Department of Neurology, Medical University of Vienna, Vienna, Austria; 3 Department of Laboratory Medicine, Division of Clinical Microbiology, Medical University of Vienna, Vienna, Austria; 4 Department of Internal Medicine III, Division of Endocrinology and Metabolism, Medical University of Vienna, Vienna, Austria; 5 Department of Emergency Medicine, Medical University of Vienna, Vienna, Austria

**Keywords:** Urinary microbiome, Kidney transplantation, Acute kidney injury (AKI), Urinary tract infection, Microbiome research

## Abstract

**Background:**

In the past urine was considered sterile. Through the introduction of next generation sequencing, it has become clear that a urinary microbiome exists. Acute kidney injury (AKI) represents a major threat to kidney transplant recipients. Remarkable changes in the urinary metabolome occur during AKI, which may influence the urinary microbiome. To our knowledge, this is the first study that examines the urinary microbiome in renal transplant recipients (RTX) and non-transplant recipients (nRTX) at time of AKI.

**Methods:**

In this cross-sectional pilot-study the urinary microbiome of 21 RTX and 9 nRTX with AKI was examined. Clean catch morning urine samples were obtained from all patients on the first day of AKI diagnosis. AKI was defined according to KDIGO guidelines. Urinary microbiota and the urinary metabolome during AKI were assessed in one patient. 16S rRNA sequencing was performed. Sequences were processed using UPARSE-pipeline for operational taxonomic units (OTU) and taxon finding.

**Results:**

We successfully extracted and sequenced bacterial DNA from 100% of the urine samples. All 30 patients revealed at least 106,138 reads. 319 OTU and 211 different genera were identified. The microbiotic diversity richness in the RTX group was no different from the nRTX group. Eighteen genera were solely present in nRTX and 7 in RTX.

**Conclusions:**

The urinary microbiome at time of AKI showed different bacterial genera in RTX compared to nRTX. The nRTX group exhibited no different diversity to the RTX group. Irrespective of the status of a previous renal transplantation, the urinary microbiome comprised > 210 different genera. An intraindividual change in microbiota diversity and richness was observed in one study patient during recovery from AKI.

## Background

Urinary tract infections (UTI) are common in renal transplant (RTX) recipients and represent a frequent cause for acute kidney injury (AKI), which may result in graft loss [1]. Urinary tract infections comprise the most common infectious complications in RTX patients, and it has been shown that untreated UTI are highly associated with acute graft rejection, dysfunction and shortened half-life of the renal graft [1–5]. Despite a long-standing misconception, urine of healthy individuals is not sterile. In the past UTI were thought to result from an invasion of pathogenic bacteria into an otherwise sterile fluid and environment. Though with the recent advances made in microbiome research, in particular with the introduction of next generation sequencing, it was discovered that dysbiosis in the urinary microbiome, such as overgrowth of a pre-existent microbe, can lead to UTI.

It is well documented in the recent literature that asymptomatic carrier stages with potentially pathogenic bacteria in urine exist, but which pose no specific threat to human health [6]. From previous research we know that a broad gamut of bacterial genera represent a steadily fluctuating flora in the urinary bladder [7–10]. These urinary bacteria have sophisticated genomic tools for keeping growth of potential bacterial competitors in a steady state and in a dynamic balance. Alongside with competition for nutrients, their genomic equipment ensures that none of the other microbial inhabitants can overgrow. Among these genomic tools are various fast responding operons, which encode polyketide synthases, cyclic lipopeptide synthases and extra-ribosomal peptide synthases, all generating biologically highly active compounds responsible for competition interference and antagonistic interaction [11–14]. These produced in a balanced mode keep urinary microbiota under healthy conditions in homeostasis. Therefore it is likely, that a disturbance of the physiological quantity and diversity of microbiota results in UTI [15–17].

To our best knowledge there are no prior studies describing the urinary microbiome of RTX compared to nRTX patients during AKI. Confronted with the above-mentioned far-reaching and harmful consequences of UTI in transplant patients in our daily clinical practice, we sought to analyze the urinary microbiome and determine if there are differences within the urinary microbiome of RTX patients compared to nRTX patients at time of AKI [3–5, 18]. In addition, we assessed for longitudinal changes of the urinary microbiome in one patient. Therefore, we decided to investigate the urinary microbiome of RTX patients with AKI, to determine if differences in the urinary microbiome of RTX and non-transplant (nRTX) patients with AKI exist. NRTX patients with AKI were selected as controls.

## Methods

### Study population

This cross-sectional study was performed at the Medical University of Vienna, Division of Nephrology and Dialysis. The study was approved by the institutional ethics committee under the number 1041/2018. Oral and written informed consent was provided by all patients before inclusion into this study. A total of thirty patients, 14 females and 16 males, were enrolled in this study on a consecutive basis. Of these, 21 represented renal transplant recipients (RTX) and 9 non-transplant patients (nRTX). All patients were admitted for AKI and had been diagnosed at the outpatient or emergency unit on the day of presentation. Patients with concurrent AKI at various stages are indicated at the demographic table (Table 1). Patients with anuria or positive history for HIV or hepatitis C virus infection were excluded from this study, because of possible confounding factors, such as the immunosuppressive potential of these viruses and antimicrobial effects of antiviral medications.

**Table 1:** Patient demographics and immunosuppressive and antibiotic regimens

	RTX (***n*** = 21)	nRTX (***n*** = 9)	***p***-value
Age, years	56 ± 16.0	62 ± 20	0.39
**Sex**			
Male	13	3	0.24
Female	8	6	0.24
**AKI stage**			
Stage 1	9	3	0.70
Stage 2	11	3	0.44
Stage 3	1	3	0.07
sCr (mg/dL)	3.0 ± 1.8	4.1 ± 3.9	0.29
BUN (mg/dL)	46.0 ± 20.7	57.8 ± 35.2	0.26
Median time after RTX (month)	5	N/A	
**Immunosuppression**			
TAC + mycophenolate+steroids	16	0	
TAC + steroids	1	0	
CYA + mycophenolate+steroids	1	0	
CYA + mycophenolate	1	0	
TAC + azathioprine+steroids	1	0	
Sirolimus+mycophenolate+steroids	1	0	
**Antibiotics**^**a**^			
Beta-lactam + beta-lactamase inhibitor	3	0	0.53
Piperacillin/tazobactam	5	1	0.64
Cephalosporin	4	0	0.29
Metronidazole	2	0	1
Fluoroquinolone	0	1	0.3

*CYA* Cyclosporine A, *eGFR* estimated glomerular filtration rate, *sCr* serum creatinine, *BUN* blood urea nitrogen, *TAC* tacrolimus, *N/A* not applicable, *±SD* standard diviation; ^a^ indicates antibiotic intake prior or at time of AKI

At the time of admission, an extensive laboratory examination was performed, which included estimated glomerular filtration rate (eGFR), serum creatinine (sCr), blood urea nitrogen (BUN), and AKI stages were defined according to KDIGO.

Out of these 30 patients, time-dependent changes in urinary microbiota of one nRTX patient were investigated for five consecutive days after diagnosis of AKI.

### Sample collection

Clean-catch midstream urine of our thirty patient sample was obtained in a sterile urine collection device. Eight milliliter urine were transferred to a Vacuette (greiner-bioone®) following a ten-minute centrifugation at 4000 g. The supernatant was discarded and the pellet was dissolved in 1000 μl TriFast™ (peqlab®) and either frozen at − 20 °C or immediately processed as described below.

### DNA isolation

The phenol-chlorophorm extraction was carried out at room temperature by adding 200 μl chlorophorm and centrifugation at 12,000 g for 5 min. The aqueous phase was narrowly taken off for RNA precipitation. Three hundred microliter of ethanol were added to the bottom layer and following repetitive inversion of the tube the DNA was pelleted by centrifugation at 2000 g for 15 min at 4 °C. The DNA pellet was then washed in 0.1Mol sodium citrate and in 10% ethanol by repetitive incubation at room temperature for 30 min. Following two washes in 75% ethanol the DNA pellet was air-dried and re-dissolved in 8mMol NaOH. The resultant solubilized DNA was neutralized using HEPES buffer solution.

### PCR amplification

The 16S rRNA gene fragment was PCR amplified using the variable 3 (V3) and variable 4 (V4) region of the 16S rRNA gene. Forward Primer and reverse primer contained Illumina® adapter overhang nucleotide sequences, which were selected according to literature precedent [19].

Purified DNA (2.5 μl) extracted from urinary sediment were used and added to 22.5 μl mastermix (KAPA HiFi HotStart Ready Mix (Roche®)) containing 16S rRNA primers 100 pmol final concentration as indicated below. The resultant mixture was then inserted into a thermal cycler. Amplification was carried out using the following conditions: 95 °C for 3 min denaturation followed by 35 cycles at 95 °C for denaturation, 52 °C for primer annealing and 72 °C for synthesis, each for 30 s. PCR product size and quantity were evaluated on a 15 lane 6% TBE NucGel (anamed electrophoresis GmbH, Germany) including DNA size markers.

### Library preparation

Twenty-five μl of the original PCR amplicon were transferred into a 96-well MIDI plate and 20 μl of premixed AMPure XP beads (Beckman Coulter, USA) were added. Following sealing of the plate and mixing on a plate shaker (1800 rpm) for 2 min and an incubation period of 5 min at room temperature, the plate was positioned on a magnetic stand for 2 min until the supernatant had cleared. The beads were then washed with 80% ethanol twice and were finally air-dried for 10 min. Fifty-five point five μL of 10 mM Tris pH 8.5 were added to each well for liberating the PCR product from the beads by incubation at room temperature for 2 min.

Five μl of the purified PCR product were transferred into a new plate containing index primer 1 and 2 and Kappa HiFi HotStart Ready Mix and were re-amplified for 8 cycles under the following conditions: 95 °C for 3 min followed by 95 °C for denaturation, 55 °C for primer annealing and 72 °C for synthesis, for 30 s respectively, followed by 5 min extension at 72 °C.

For final clean-up, 56 μL of AMPure XP beads were added to each well and DNA amplicons were bound to the beads. All washing procedures were performed in a similar manner as described above for purification of the primary PCR product. The indexed amplicons were liberated from the beads by 27.5 μL of 10 mM Tris pH 8.5 and each sample was subjected to quantification and validation.

The Quanti-iT™ dsDNA Broad-Range Assay Kit (molecular probes, life technologies) was utilized for DNA-quantification of each individual purified sample and equimolar ratios were pooled and applied into the Illumina MiSeq sequencing device. The sequencing machine was run with the Version 3 of the Illumina MiSeq program. Samples were run in several batches of 24 samples per run.

### Sequence processing and taxonomic assignment

Purified and quantitated bacterial V3, V4 regions’ DNA were sequenced using the Illumina MiSeq Device (Version 3). Next generation sequencing (NGS) data were trimmed using trimmomatic to get paired reads with length of at least 100 bases [20]. For OTU generation we used the UPARSE-pipeline with chimera filtering quality filtering and a cut-off of 97% similarity [21]. As taxonomic database we used the 16S-rdp-database (release 16) and for comparison and statistical analysis of the microbiomes we utilized RStudio (v1.1.463) as platform with R as language (v3.5.1) with different R-packages [22–24]. Taxonomic information (sintax) and the OTUtable were imported into RStudio by means of devtools and RDPutils, the reads were rarefied to 106.138 or 215.323 for RTX/nRTX-comparison or longitudinal analysis (vegan) respectively. Statistical analysis were performed by means of ape and phyloseq [25].

Diversity measures were calculated using Chao1, Shannon and the inverse Simpson method. Detailed analysis was performed in RStudio (Version 1.1.463) [26–28].

### Metabolome analysis

Urine samples from days one to five were subjected to a methanol extraction step to remove protein. The supernatant was subsequently analyzed by LC-MS using a ZIC-pHILIC column on an Ultimate 3000 RSLC system linked to a Thermo Fisher Scientific q-Exactive instrument (m/z detection range 60–900). Compound Discoverer 3.0 software was used for identification. An internal database and mzCloud database were applied. Analysis was restricted to 288 compounds identified as follows: 52 compounds were identified by matching retention time and exact mass (5 ppm tolerance) to a previously measured internal standard sample of known compounds, in case an MS2 spectrum was available this was also taken into account. Further 236 compounds were identified by matching exact mass (5 ppm tolerance) and MS2 spectrum to the mzCloud database. Note that a repeated detection of a compound with highly similar exact mass but at a different retention time may reflect for instance isomers (e.g. stereo isomers or structural isomers with similar fragmentation pattern) or additional peaks usually eluting later during the elution profile of a substance. For further data analysis, all areas were log2 transformed, and an average of the two replicates was calculated for days one to five respectively (corresponding to the log2 transformation of the geometric mean). Subsequently a linear regression model based on the least squares method was calculated for each compound (with 1 to 5 as x-values), and the list of identified compounds was sorted according to decreasing parameter m. Thus, compounds at the top of the list are those for which the model indicates the most pronounced increase, whereas compounds at the bottom of the list are those for which the model indicates the most pronounced decrease. The coefficient of determination R2 was also calculated as a measure of the fit between the linear model and the measured values. Finally, the log2-transformed ratio of the geometric means on day 5 to day 1 was calculated, as a measure of changes from the first to the last day of the observation period.

### Statistical analysis

Adherence to a Gaussian distribution was determined using the Kolmogorov-Smirnov test. Normally distributed data were described as means ± standard deviations. In case of skewed distribution data were described as medians (25th and 75th percentiles). Qualitative variables were described with counts and percentages and compared using Fisher’s exact test. A two-tailed *p*-value of 0.05 was considered statistical significant. Data were analyzed with SPSS® Statistics (Version 21 for Mac).

## Results

### Study population and patient demographics

Twenty-one RTX patients and 9 nRTX patients without clinical signs or symptoms of UTI were included directly after onset of AKI.

In all 9 nRTX patients AKI was the reason for admission. Reasons for AKI in the nRTX group were intestinal fluid loss in two patients, drug induced AKI in one patient and rhabdomyolysis, tumor lysis syndrome, and cardiorenal syndrome in two patients respectively.

In the RTX group, nine patients experienced AKI stage 1 according to KDIGO, 11 patients suffered from stage 2 and one patient from AKI stage 3. In the nRTX group 3 patients experienced AKI stages 1, 2 and 3, respectively. There were no statistical significant differences in the severity of AKI between RTX and nRTX patients (Table 1). Estimated glomerular filtration rate (eGFR), serum creatinine (sCr) and blood urea nitrogen (BUN) levels at the date of microbiome evaluation were investigated and did not differ between the RTX and the nRTX group (Table 1).

Out of the 21 RTX patients 16 received a triple immunosuppressive regimen with tacrolimus (TAC), mycophenolate and corticosteroids or azathioprine and steroids. Deviating immunosuppressive therapy schemes of study patients are shown in Table 1.

Renal transplant and non-transplant patients did not differ in the assessed distribution of covariates except for intake of immunosuppressive drugs (*p* < 0.001).

As several previous urinary microbiome studies could not confirm the presence of bacteria in 100% of urine samples, the first step was to verify the existence of a urinary bacterial microbiome in our study population. Therefore, urinary DNA was isolated and 16S rRNA sequencing was performed, and the bacterial DNA load within the urine of RTX and nRTX patients with AKI was determined.

### The urine of RTX and nRTX patients with AKI comprises a rich microbiome

**Fig. 1:**
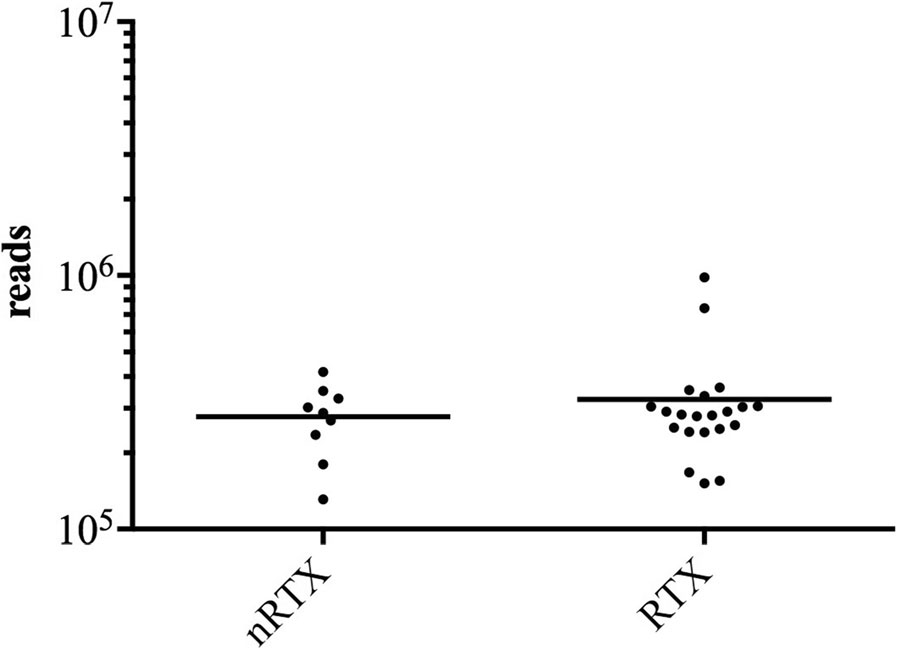
Absolute read count of bacterial V3/V4 region DNA-sequence in urine from 9 nRTX and 21 RTX patients with AKI. Urine of RTX AKI patients comprised a DNA load of 325,588 ± 191,717, while those nRTX AKI patients exhibited 278,026 ± 87,261 reads. No statistically significant difference in bacterial DNA load in the urine of RTX and nRTX AKI patients was detected (*p* = 0.48). None of the tested samples exhibited sequencing reads below 106,138 reads

We extracted and sequenced bacterial DNA from 100% of urine samples. The absolute bacterial V3/V4 region read count was variable and depended on the individual patient. The mean read count in the RTX group was 325,588 ± 191,717 and in the nRTX group 278,026 ± 87,261, and showed no significant difference between the groups (*p* = 0.48) (Fig. 1). None of the participants showed read numbers below 106,138.

Hence, urine of all included RTX and nRTX patients comprised no different bacterial DNA reads, indicating the presence of a rich urinary microbiome in RTX and nRTX patients with AKI. This led to the next step of determining the exact bacterial genera and to classify closely related bacteria via taxon finding.

### The urinary microbiome exhibits different genera in RTX and nRTX AKI patients

Taxon finding was performed to classify the groups of closely related bacteria and investigate their presence in RTX and nRTX with AKI.

For operational taxonomic unit (OTU) generation, bacterial reads of all patients were rarified and loaded into UPARSE-pipeline with a cut-off of 97%, which identified 319 OTU. Of these, genus taxon levels could be delineated for 211 OTU, which were then investigated for the prevalence among RTX and nRTX AKI patients.

**Fig. 2:**
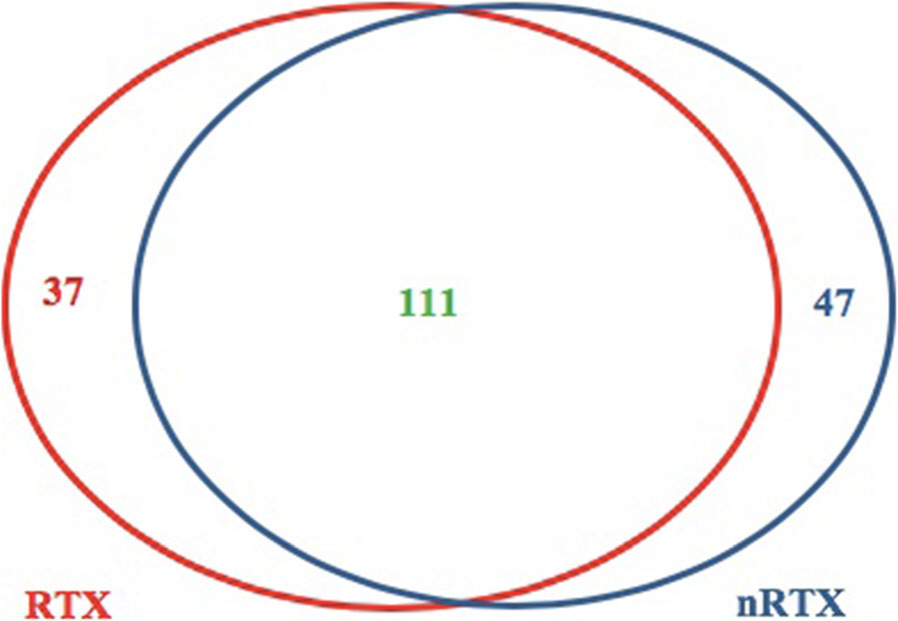
Venn diagram of urinary bacterial genera. RTX and nRTX AKI patients had an overlap of 111 genera, 37 genera were found exclusively among RTX and 47 genera solely in nRTX patient

RTX and nRTX shared 111 common bacterial genera. In total 47 bacterial genera could be found only in the RTX group, while 37 genera were solely present in the nRTX group (Fig. 2).

Hence, there appeared to be bacterial genera which were either found in the RTX or in the nRTX group, which could be of clinical relevance. However some of them could only be detected with a very small DNA load and low prevalence, especially in the RTX group, which made us suspect that these might not be of clinical importance. Therefore, we decided to investigate bacterial genera with a prevalence > 25% in either the RTX or the nRTX group.

**Fig. 3:**
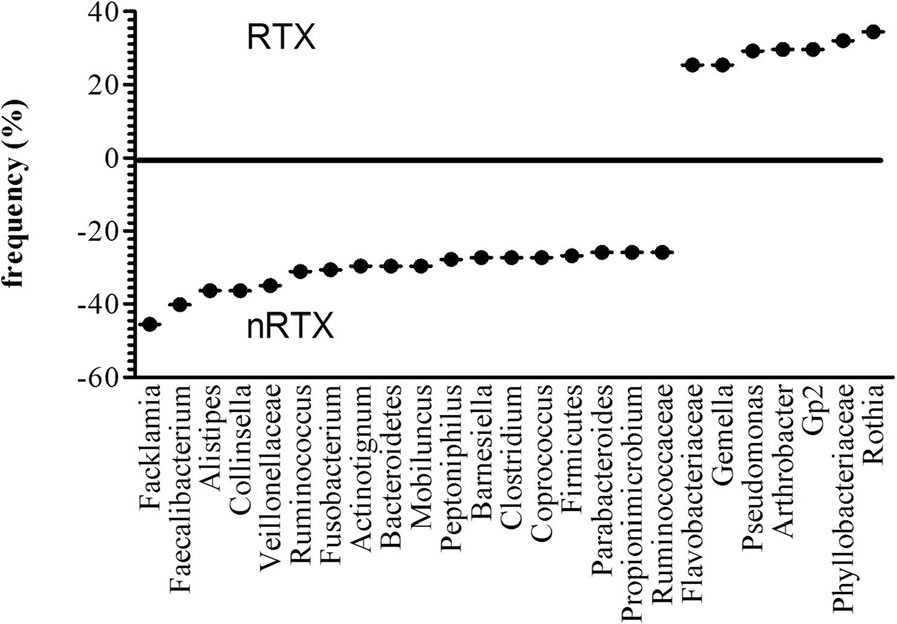
Prevalence of urinary bacterial genera in RTX and nRTX AKI patients. A cut-off of 25% was chosen, therefore only prevalence of bacterial taxa with either > 25% higher or < 25% prevalence is depicted. Marks above 0 give higher prevalence in RTX urine, marks below 0 give higher prevalence in nRTX

Seven bacterial taxa (*Flavobacteriaceae, Gemella, Pseudomonas, Arthrobacter, Gp2, Phyllobacteriaceae, Rothia*) were found to be present with a prevalence greater 25% only in RTX AKI patients compared to nRTX patients. In contrast, 18 taxa (*Facklamia, Faecalibacterium, Alistipes, Collinsella, Veillonellaceae, Ruminococcus, Fusobacterium, Actinotignum, Bacteroidetes, Mobiluncus, Peptoniphilus, Barnesiella, Clostridium, Coprococcus, Firmicutes, Parabacteroides, Propionimicrobium, Ruminococcaceae*) were found with a prevalence of > 25% only in nRTX patients (Fig. 3).

Thus, it appears that there are bacteria which are more likely to inhabit the urinary tract of RTX AKI patients which are not found in nRTX AKI patients and vice versa. The bacterial genera mentioned above were present in > 25% of patients of one group and therefore were likely to be present in high numbers in urine. However, it has been described in previous literature that not only single prevalent bacteria, but the overall diversity of the human microbiome is likely to be associated with health and disease [17, 29, 30]. Therefore, it is of interest weather the overall diversity of the urinary microbiome is different between RTX and nRTX AKI patients.

### The overall diversity of the urinary microbiome in RTX and nRTX AKI patients is similar

It has been presented in prior research that the overall microbial diversity preserves a healthy environment in the urinary tract [29, 30]. For this reason the diversity measures Chao1, Shannon and Inverse Simpson were calculated. As depicted in Table 2, all three diversity indices tended towards a higher diversity in the nRTX group than in the RTX group (Chao1 536.02 ± 641.97 vs. 383.02 ± 232.73; Shannon 2.83 ± 1.31 vs. 2.39 ± 1.01; Inverse Simpson 12.83 ± 17.43 vs. 7.73 ± 7.66). However, none of these three diversity measures exhibited statistically significant differences with a *p*-value of 0.18, 0.15, and 0.14 respectively. The taxonomic beta diversity data can be found in Additional file 1.

**Table 2:** Diversity richness calculation using Chao1, Shannon and inverse Simpson and calculated p-levels

	RTX	nRTX	***p***-value
**Chao1**	383.02 ± 232.73	536.02 ± 641.97	0.18
**Shannon**	2.39 ± 1.01	2.83 ± 1.31	0.15
**InvSimpson**	7.73 ± 7.66	12.83 ± 17.43	0.14

*InvSimpson* Inverse Simpson, *RTX* renal transplant recipients, *nRTX* non-renal transplant recipients

Data are given as mean ± standard deviation with *p*-values derived from independent sample t-test

In summary, there was no statistically significant difference of the microbiome diversity in nRTX and RTX AKI patients.

In addition, as part of the pilot project it was considered important to investigate the diurnal changes of the urinary microbiome within a single individual, especially during regain of kidney function.

### Urinary microbiome changes occurred during AKI recovery in a single patient

To investigate if the urinary microbiome was altered during recovery from AKI, urinary microbiota were analyzed on a daily basis in the phase of serum creatinine decline. Therefore, a 52-years old, male nRTX patient who recovered from diarrhea-induced AKI stage 3 was randomly selected and daily urine samples were obtained and immediately analyzed. The patient did not receive antibiotics prior or during the study period.

**Fig. 4:**
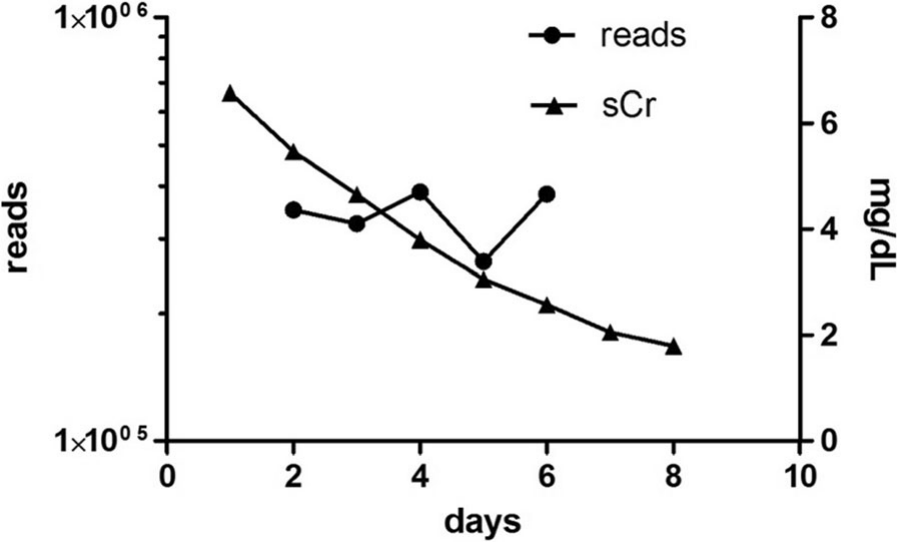
Five-day follow up of the prokaryotic DNA load in urine and kidney function of a 52-years old nRTX patient recovering from AKI. During decrease of serum creatinine levels, the bacterial read count stayed approximately the same. Kidney function is given as serum creatinine (sCR) and DNA load is measured in reads

The bacterial read count of urinary bacteria varied between 260,000 and 380,000 and was independent of the status of renal function **(**Fig. 4).

**Fig. 5:**
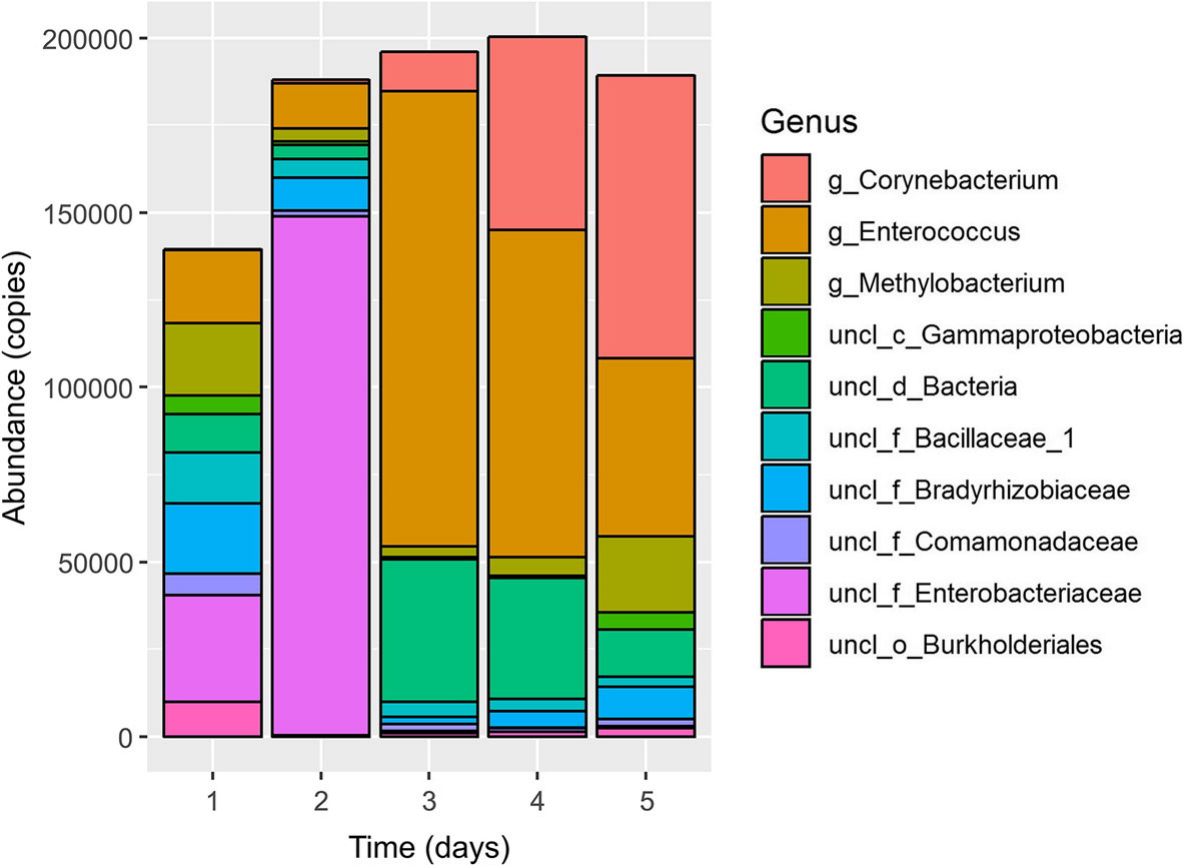
Intraindividual changes in the urinary microbiome of a 52-years old nRTX patient during regain of kidney function. The 10 most abundant genera occurring during this time period are depicted. Daily shifts and variations within the urinary microbiome could be observed. While on the first day’s measurement a more or less balanced distribution of the 10 most abundant genera is outlined, on the second day expansion of *Enterobacteriaceae* eventuated, which nearly vanished on day three in favor for *Enterococcus*. On day four and five, while kidney function was gradually improving, it appeared that a new relative balance favoring *Enterococcus*, *Corynebacterium* and *Methylobacterium* occurred

In this longitudinal experiment, 115 OTU at the genus level were identified throughout the observation period of which 70 genera underwent a change of more than 30% within 5 days. Figure 5 depicts the 10 most abundant genera occurring during recovery from AKI. The patient did not receive any antibiotic treatment and did not show any symptoms of urinary tract infection during a follow-up period of 14 days.

Therefore it appeared that the presence of a urinary microbiome was not dependent on the urine volume as the quantity of produced urine changed during recovery from AKI. In addition, changes within the urinary microbiome during recovery from AKI occurred on an individual patient level and we assume that these changes were associated with changes in the urinary metabolome.

### Intraindividual changes within the urinary metabolome occur during AKI recovery in a single patient

As indicated above, our data suggested differences in the urinary microbiome during improvement of kidney function in one patient. As has been shown earlier, the urinary metabolome experiences changes during recovery from AKI [31]. This led to the hypothesis that during this time period shifts within the metabolome might occur and represent a modulator for changes in the microbiome. Therefore we investigated whether changes in the urinary metabolome occurred in the same patient in whom the diurnal urinary microbiome was investigated.

Therefore, we performed an untargeted metabolomics investigation of urine collected on days one to five during AKI. The resulting list of compounds was sorted such that compounds that increased most strongly during the observation period (based on a linear regression model) were listed at the top, where compounds that decreased most strongly were listed at the bottom (Additional file 2). Results indicate alterations in the abundance of several metabolic compounds during the first days after AKI, such as for instance a significant increase in methylsuccinic acid, succinic acid, hypoxanthine, xanthosine, ethylmalonic acid, methylguanine, lactic acid, hydroxyglutaric acid, oxoglutaric acid, isoleucine, lactose, citrulline, histidine, uracil, asparagine, alanine, and a decrease in iditol, mannitol and ornithine.

### Limitations of the study

Limitations of this pilot study include the small sample size. Unfortunately, we were only able to obtain daily follow-up samples of one patient. The cross-sectional study design represents a further limitation as patients were not matched on AKI stages, types of AKI, urine output on day of sample collection, and immunosuppressive regimen, which could have had an impact on the urinary microbiota of RTX patients. Measurement of overall urine output was not available in all patients. In addition, although microbiome research is booming, microbial genomic datasets are still not entirely complete, albeit nearly-complete and reproducibility strongly depends on the database utilized [8]. Furthermore potential technical limitations associated with metagenomics and amplicon sequencing remain, which have already been extensively discussed in former literature. These include sampling errors, primer or processing biases, and the use of adequate analytical algorithms. Moreover identification of additive, synergistic or antagonistic effects remains challenging [32–35].

## Discussion

Kidney transplant recipients have an elevated AKI risk, which may result in graft loss [36, 37]. Urinary tract infections are the most prevalent infectious complication in RTX patients and play a major cause in the development of AKI [1]. Hence, it is important to ascertain whether the urinary microbiome in RTX patients differs from those of nRTX patients at time of AKI. We were able to detect a rich and diverse urinary microbiome in all included patients. Different bacterial genera could be detected in copious numbers in either RTX or nRTX only. The overall bacterial diversity was equal in nRTX and RTX AKI patients. Finally our single patient data suggest that the intraindividual urinary microbiome might undergo daily changes during the period of kidney function regain.

In reference to the existence of a microbiome in the patients’ urine, all RTX and all nRTX AKI patients exhibited equally high numbers of bacterial DNA in their urine. These data substantiate that urine is not sterile and has a diverse bacterial environment, as has been extensively documented in recent years [7, 8, 10, 29]. In addition, our study confirmed data published by Fricke et al. that bacterial communities are abundantly present in urine of RTX patients [38].

Regarding the bacterial richness, taxon finding was performed. Therefore we decided to analyze urinary bacteria on a genus level rather than on a family or phylum level, which is commonly done in microbiome research as analyses on a higher taxonomic level yield the advantage of increasing the chances for statistically significant results. However, reasons for the decision to analyze our data on a genus-level were based on clinical aspects, as hierarchal higher taxonomic levels aggregate a great number of bacteria but state little about the actual microbial flora. As a matter of fact, it can be argued that even with an analysis on a genus-level an uncertainty about the underlying bacteria remains. As an example, the genus *Clostridium* contains about 100 species, which exhibit different growth characteristics and are either harmless to humans or responsible for various diseases [39]. For instance, *Clostridium botulinum* produces the lethal botulinum toxin, which causes botulism [40]. Other species from the genus *Clostridium* include *Clostridium tetani*, the causative agent of tetanus, *Clostridium perfringens*, which causes gas gangrene, *Clostridium difficile*, the cause of diarrhea [41–43]. Unfortunately we could not analyze bacteria on a species-level as the V3 and V4 regions which are detected with 16S rRNA sequencing are highly preserved.

Nevertheless, the question arises why some genera were only present in RTX and not in nRTX AKI patients and vice versa. As mentioned above, except for the transplant status and the intake of immunosuppressive drugs, there were no statistical significant differences between the two groups (Table 1). Therefore there are three possible hypotheses:

(i) Immunosuppressants either directly inhibit growth of certain bacterial genera, while other bacteria are not or less effected, or immunosuppression indirectly stimulate growth of specific bacteria [44]. (ii) Iatrogenic anatomical differences leading to changed urodynamics between RTX and nRTX patients can influence the composition of the microbiome. This occurs as renal transplants mostly possess a shortened ureter, which leads into a shrunken urinary bladder, which has an effect on urine outflow when compared to nRTX patients [45–48]. (iii) The urinary metabolome differs in RTX and nRTX patients and it is likely that the urinary metabolite profile has a growth enhancing or growth inhibitory effect on certain bacterial genera [49]. (iv) In addition, a difference in the urinary microbiome of RTX and nRTX patients may occur due to frequent healthcare contacts of nRTX patients. We believe all four possibilities are equally likely to apply.

Referring to the daily intraindividual dynamics in the urinary microbiome of an nRTX patient recovering from AKI, we hypothesize that also these shifts depended on metabolite and electrolyte excretion in urine during the recovery from AKI, as it has already been well documented in recent literature that the urinary metabolome experiences changes dependent on kidney function [49, 50]. This is consistent with our metabolome analysis of this patient. Another possible explanation for these intraindividual alterations is competitive growth of the bacteria themselves. When one genus is absent, or only present in small numbers, another genus is able to grow out and fill the niche. This could be facilitated by bioactive compounds in urine produced as a bacterial machinery of defense against other bacteria. As mentioned above, such antimicrobial products include polyketide synthases, cyclic lipopeptidases and extra-ribosomal peptide synthases [11–13]. Whether the production of antimicrobial products varies during AKI has not been investigated so far and needs to be evaluated in future studies.

## Conclusion

In conclusion, our pilot study is the first comparing the urinary microbiome of RTX and nRTX patients at time of AKI. Our data demonstrated that all included patients possessed a rich and diverse microbiome with significant differences regarding the occurrence of certain bacterial genera between RTX and nRTX, while overall diversity did not differ between the two groups. In addition, an intraindividual change in urinary microbiota during recovery of kidney function was observed in a single patient and might reflect coinciding changes in the metabolome also documented in this work.

## Supplementary information

**Additional file 1.** Beta diversity of urinary microbiota of RTX and nRTX AKI patients calculated with Shannon divergence (left) and Pearson correlation (right). Several samples were clustering. However, no particular clustering of RTX and nRTX groups could be observed.

**Additional file 2.** Metabolome analysis of a 52-year-old nRTX patient during a 5-day period of recovery from AKI. Urinary compounds that increased during recovery are listed at the top, while compounds that decreased are listed at the bottom.

## Data Availability

The datasets generated and analyzed during the current study are available from the corresponding author on reasonable request.

## Abbreviations

AKI: Acute kidney injury
BUN: Blood urea nitrogen
DNA: Deoxyribonucleic acid
dsDNA: Double-stranded deoxyribonucleic acid
eGFR: Estimated glomerular filtration rate
KDIGO: Kidney Disease Improving Global Outcomes
NaOH: Sodium hydroxide
nRTX: Non-renal transplant
OTU: Operational taxonomic unit
PCR: Polymerase chain reaction
rRNA: Ribosomal ribonucleic acid
RTX: Renal transplant
sCr: Serum creatinine
UTI: Urinary tract infection
