# Correction to: The urinary microbiome shows different bacterial genera in renal transplant recipients and non-transplant patients at time of acute kidney injury – a pilot study

**DOI:** 10.1186/s12882-020-01835-4

**Published:** 2020-05-07

**Authors:** Daniela Gerges-Knafl, Peter Pichler, Alexander Zimprich, Christoph Hotzy, Wolfgang Barousch, Rita M. Lang, Elisabeth Lobmeyr, Sabina Baumgartner-Parzer, Ludwig Wagner, Wolfgang Winnicki

**Affiliations:** 1 Department of Internal Medicine III, Division of Nephrology and Dialysis, Medical University of Vienna, Waehringer Guertel 18-20, 1090 Vienna, Austria; 2 Department of Neurology, Medical University of Vienna, Vienna, Austria; 3 Department of Laboratory Medicine, Division of Clinical Microbiology, Medical University of Vienna, Vienna, Austria; 4 Department of Internal Medicine III, Division of Endocrinology and Metabolism, Medical University of Vienna, Vienna, Austria; 5 Department of Emergency Medicine, Medical University of Vienna, Vienna, Austria

**Correction to: BMC Nephrology (2020) 21:117**

**https://doi.org/10.1186/s12882-020-01773-1**

Following publication of the original article [1], we were notified that an author’s first and last name had been reversed.

Incorrect: Pichler Peter

Correct: Peter Pichler

The original article has been corrected.
