# Supplementary material for: The urinary microbiome shows different bacterial genera in renal transplant recipients and non-transplant patients at time of acute kidney injury – a pilot study
Source: BMC Nephrol. 2020 Apr 6;21:117. doi: 10.1186/s12882-020-01773-1 (PMC7133001; doi:10.1186/s12882-020-01773-1)

**Additional File 1. Beta diversity of urinary microbiota of RTX and nRTX AKI patients calculated with Shannon divergence (left) and Pearson correlation (right).** Several samples were clustering. However, no particular clustering of RTX and nRTX groups could be observed.


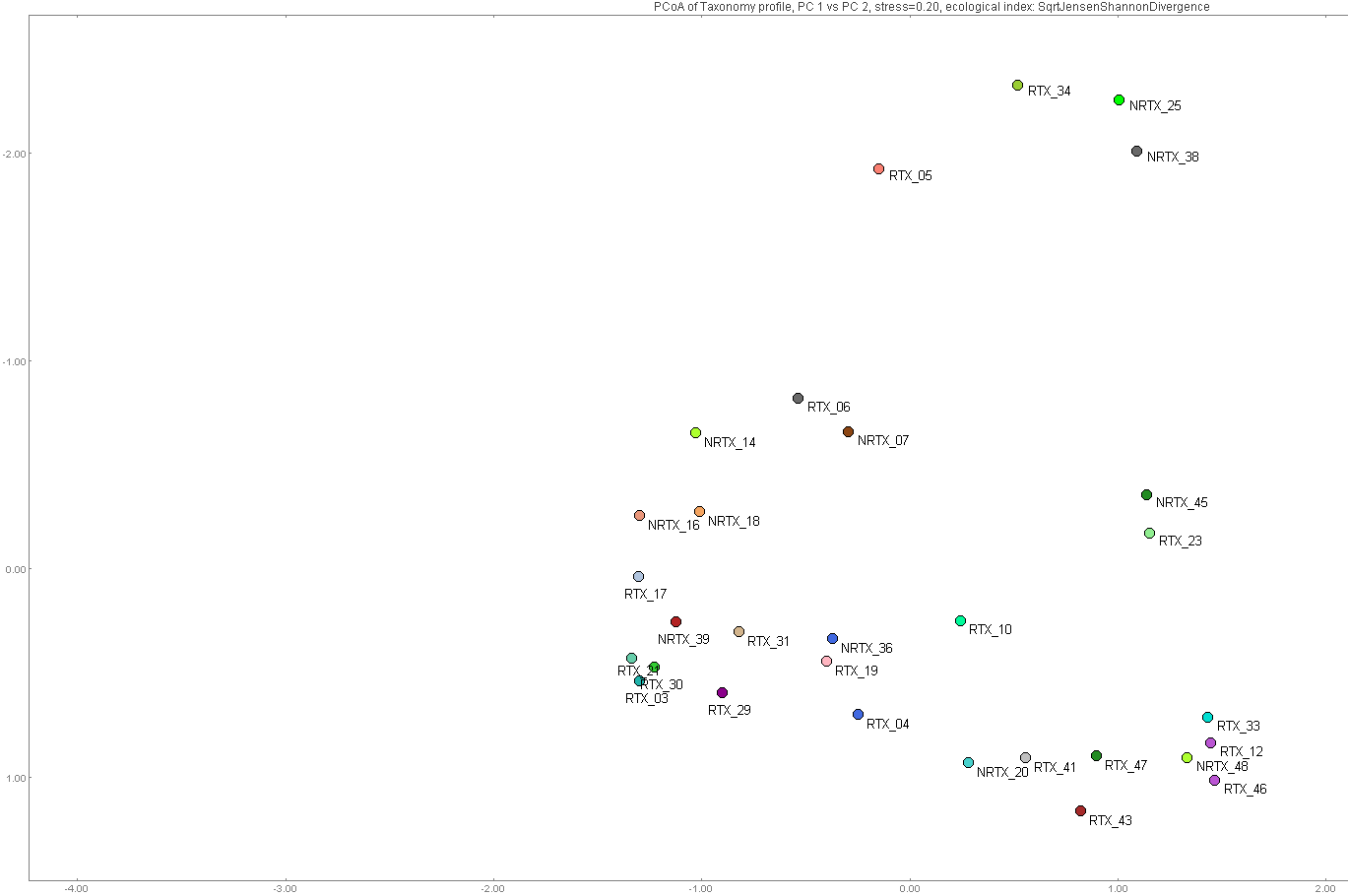

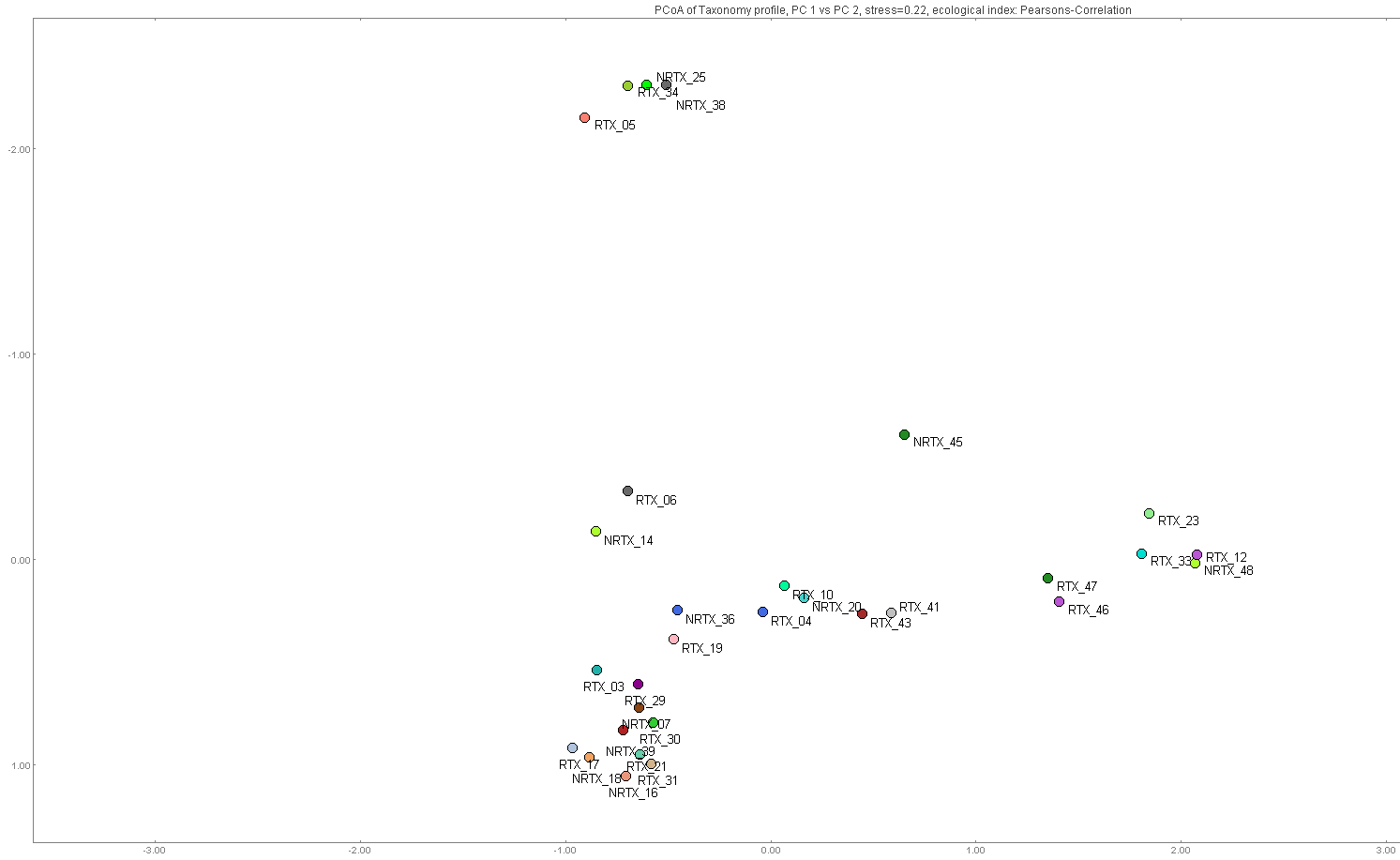

Supplement: Supplementary file 1 — Additional file 1. Beta diversity of urinary microbiota of RTX and nRTX AKI patients calculated with Shannon divergence (left) and Pearson correlation (right). Several samples were clustering. However, no particular clustering of RTX and nRTX groups could be observed. [file 12882_2020_1773_MOESM1_ESM.docx]
