# Supplementary material for: The urinary microbiome shows different bacterial genera in renal transplant recipients and non-transplant patients at time of acute kidney injury – a pilot study
Source: BMC Nephrol. 2020 Apr 6;21:117. doi: 10.1186/s12882-020-01773-1 (PMC7133001; doi:10.1186/s12882-020-01773-1)
Supplement: Supplementary file 2 — Additional file 2. Metabolome analysis of a 52-year-old nRTX patient during a 5-day period of recovery from AKI. Urinary compounds that increased during recovery are listed at the top, while compounds that decreased are listed at the bottom. [file 12882_2020_1773_MOESM2_ESM.docx]

**Additional File 2. Metabolome analysis of a 52-year-old nRTX patient during a 5-day period of recovery from AKI.** Urinary compounds that increased during recovery are listed at the top, while compounds that decreased are listed at the bottom.

| **Name** | **mass [Da]** | **RT [min]** | **log2 d1** | **log2 d2** | **log2 d3** | **log2 d4** | **log2 d5** | **linest m** | **linest R2** | **log2 (d5:d1)** |
| --- | --- | --- | --- | --- | --- | --- | --- | --- | --- | --- |
| 2-Hydroxyhippuric acid | 195.0532 | 6.51 | 17.97 | 19.20 | 19.25 | 25.90 | 25.81 | 2.24 | 0.83 | 7.84 |
| Benzoic acid | 122.0369 | 5.95 | 16.14 | 16.81 | 18.64 | 23.51 | 23.39 | 2.12 | 0.89 | 7.25 |
| 1.5-Anhydro-D-glucitol | 164.0687 | 9.92 | 17.45 | 21.55 | 23.34 | 23.96 | 24.23 | 1.60 | 0.81 | 6.78 |
| Methylsuccinic acid | 132.0424 | 15.91 | 13.98 | 15.05 | 15.34 | 19.31 | 19.40 | 1.51 | 0.88 | 5.42 |
| 3.4-Dihydroxybenzenesulfonic acid | 189.9937 | 2.44 | 21.07 | 21.51 | 23.73 | 25.59 | 25.52 | 1.30 | 0.92 | 4.45 |
| cis.cis-Muconic acid | 142.0268 | 7.11 | 15.70 | 16.74 | 18.12 | 18.46 | 20.95 | 1.22 | 0.94 | 5.25 |
| (S)-Nicotine | 162.1158 | 2.87 | 16.84 | 18.16 | 18.66 | 18.10 | 22.95 | 1.22 | 0.68 | 6.11 |
| Cholic acid | 408.2880 | 2.88 | 14.46 | 14.37 | 14.55 | 17.53 | 18.53 | 1.13 | 0.81 | 4.06 |
| δ-Gluconic acid δ-lactone | 178.0479 | 8.80 | 17.54 | 18.11 | 20.14 | 20.48 | 20.93 | 0.92 | 0.91 | 3.40 |
| 2-Hydroxyhippuric acid | 195.0532 | 2.95 | 18.54 | 20.79 | 20.60 | 21.87 | 22.49 | 0.90 | 0.88 | 3.95 |
| 4-Anisic acid | 152.0475 | 2.64 | 21.55 | 22.13 | 20.47 | 25.01 | 24.53 | 0.88 | 0.51 | 2.98 |
| trans-Zeatin | 219.1120 | 8.72 | 17.66 | 18.02 | 19.82 | 20.80 | 20.57 | 0.86 | 0.88 | 2.91 |
| D-Alanyl-D-alanine | 160.0849 | 8.05 | 17.41 | 18.94 | 21.29 | 20.23 | 20.95 | 0.84 | 0.69 | 3.54 |
| 5-Sulfosalicylic acid | 217.9886 | 17.27 | 21.27 | 21.57 | 21.21 | 23.32 | 24.54 | 0.83 | 0.78 | 3.27 |
| Phenol | 94.0419 | 2.64 | 17.43 | 19.31 | 17.56 | 21.11 | 20.66 | 0.83 | 0.59 | 3.23 |
| N-Acetyl-L-leucine | 173.1053 | 2.65 | 15.91 | 17.13 | 20.00 | 18.35 | 19.41 | 0.82 | 0.61 | 3.50 |
| Acetaminophen glucuronide | 327.0957 | 12.41 | 15.55 | 16.55 | 17.50 | 18.37 | 18.24 | 0.72 | 0.91 | 2.69 |
| N-Acetyltyramine | 179.0947 | 2.30 | 16.64 | 17.83 | 19.07 | 19.59 | 19.34 | 0.72 | 0.84 | 2.70 |
| 5'-S-Methyl-5'-thioadenosine (MTA) | 297.0896 | 4.64 | 16.39 | 15.86 | 17.31 | 16.94 | 19.02 | 0.63 | 0.69 | 2.62 |
| Hypoxanthine | 136.0386 | 9.14 | 20.66 | 21.55 | 21.33 | 23.38 | 22.70 | 0.59 | 0.72 | 2.04 |
| 2-Aminoadipic acid | 161.0689 | 7.57 | 17.87 | 19.60 | 20.18 | 20.34 | 20.36 | 0.57 | 0.74 | 2.49 |
| Succinic acid | 118.0267 | 20.21 | 16.63 | 17.74 | 18.34 | 18.29 | 19.21 | 0.57 | 0.91 | 2.58 |
| 1-Aminocyclohexanecarboxylic acid | 143.0948 | 19.20 | 20.75 | 21.39 | 21.49 | 22.56 | 22.94 | 0.56 | 0.95 | 2.20 |
| DL-4-Hydroxyphenyllactic acid | 182.0581 | 5.87 | 24.03 | 24.30 | 23.75 | 25.93 | 25.92 | 0.54 | 0.65 | 1.89 |
| Vanillic acid | 168.0424 | 6.61 | 21.73 | 22.79 | 20.46 | 23.82 | 23.91 | 0.54 | 0.34 | 2.19 |
| Paraxanthine | 180.0647 | 9.46 | 19.24 | 20.89 | 21.29 | 21.54 | 21.58 | 0.53 | 0.75 | 2.34 |
| 4-Guanidinobutyric acid | 145.0852 | 16.15 | 19.19 | 19.47 | 21.08 | 20.42 | 21.27 | 0.51 | 0.75 | 2.08 |
| Glycolic acid | 76.0161 | 7.53 | 19.89 | 20.07 | 21.50 | 21.11 | 21.86 | 0.50 | 0.82 | 1.97 |
| Triethanolamine | 149.1053 | 7.63 | 18.02 | 18.43 | 18.29 | 19.98 | 19.61 | 0.47 | 0.74 | 1.58 |
| 3.4-Dihydroxyphenylpropionic acid | 182.0581 | 3.03 | 20.30 | 20.56 | 20.28 | 21.72 | 21.96 | 0.45 | 0.76 | 1.66 |
| Xanthosine | 284.0758 | 11.55 | 18.59 | 19.32 | 19.30 | 20.20 | 20.37 | 0.44 | 0.92 | 1.78 |
| trans-3-Hydroxycotinine | 192.0900 | 4.00 | 19.63 | 19.73 | 20.37 | 20.73 | 21.24 | 0.42 | 0.97 | 1.62 |
| 2-Furoylglycine | 169.0376 | 2.99 | 19.08 | 20.77 | 21.06 | 20.58 | 21.27 | 0.42 | 0.59 | 2.18 |
| Ethylmalonic acid | 132.0422 | 4.12 | 20.55 | 21.19 | 21.63 | 22.00 | 22.19 | 0.41 | 0.96 | 1.63 |
| Citraconic acid | 130.0268 | 16.58 | 19.11 | 19.69 | 19.86 | 20.38 | 20.65 | 0.38 | 0.98 | 1.54 |
| 7-Methylguanine | 165.0652 | 8.79 | 23.16 | 23.72 | 24.15 | 24.57 | 24.61 | 0.37 | 0.94 | 1.45 |
| DL-Lactic Acid | 90.0318 | 4.74 | 22.39 | 22.27 | 23.24 | 23.15 | 23.78 | 0.37 | 0.85 | 1.40 |
| Succinic acid | 118.0267 | 15.90 | 20.54 | 20.87 | 20.88 | 21.45 | 22.07 | 0.36 | 0.91 | 1.53 |
| 2-Furoylglycine | 169.0376 | 4.56 | 20.54 | 21.83 | 22.01 | 21.67 | 22.42 | 0.36 | 0.66 | 1.88 |
| L-Homoserine | 119.0584 | 15.88 | 18.54 | 18.93 | 19.66 | 19.85 | 19.85 | 0.35 | 0.88 | 1.31 |
| Tropine | 141.1155 | 21.55 | 16.57 | 15.81 | 17.90 | 16.42 | 18.03 | 0.35 | 0.33 | 1.46 |
| N-Acetylputrescine | 130.1107 | 22.48 | 22.34 | 23.25 | 23.55 | 23.71 | 23.87 | 0.35 | 0.84 | 1.53 |
| D-α-Hydroxyglutaric acid | 148.0373 | 15.11 | 24.55 | 25.01 | 25.31 | 25.92 | 25.85 | 0.35 | 0.93 | 1.31 |
| (-)-Camphanic acid | 198.0893 | 8.80 | 20.59 | 21.15 | 21.57 | 21.98 | 21.93 | 0.35 | 0.91 | 1.34 |
| 4-Phenolsulfonic acid | 173.9987 | 2.23 | 23.26 | 23.27 | 23.99 | 24.42 | 24.45 | 0.35 | 0.90 | 1.18 |
| DL-Lactic Acid | 90.0318 | 3.04 | 20.10 | 19.96 | 20.93 | 20.56 | 21.55 | 0.35 | 0.74 | 1.45 |
| Isobutyric acid | 88.0525 | 4.12 | 20.18 | 20.66 | 20.99 | 21.38 | 21.55 | 0.35 | 0.98 | 1.38 |
| N-Acetylvaline | 159.0896 | 3.03 | 20.67 | 20.68 | 20.53 | 21.81 | 21.84 | 0.35 | 0.70 | 1.17 |
| N2-Methylguanosine | 297.1073 | 9.75 | 20.71 | 21.28 | 21.42 | 21.98 | 22.09 | 0.35 | 0.95 | 1.38 |
| Bisoprolol | 325.2252 | 6.41 | 22.72 | 23.64 | 23.90 | 24.48 | 24.00 | 0.34 | 0.68 | 1.28 |
| Adipic acid | 146.0580 | 15.01 | 17.66 | 18.37 | 19.08 | 19.04 | 19.02 | 0.34 | 0.75 | 1.36 |
| 2-Aminonicotinic acid | 138.0430 | 4.54 | 19.45 | 19.24 | 20.01 | 19.95 | 20.76 | 0.33 | 0.80 | 1.31 |
| 2-Oxoglutaric acid | 146.0217 | 16.75 | 23.04 | 23.59 | 23.49 | 24.21 | 24.37 | 0.33 | 0.90 | 1.32 |
| D-α-Hydroxyglutaric acid | 148.0373 | 16.58 | 22.47 | 22.99 | 23.11 | 23.68 | 23.76 | 0.33 | 0.95 | 1.28 |
| Succinic semialdehyde | 102.0318 | 16.75 | 20.38 | 20.97 | 20.88 | 21.59 | 21.70 | 0.33 | 0.90 | 1.32 |
| 3-Methylhippuric acid | 193.0740 | 2.54 | 19.37 | 19.39 | 20.21 | 19.98 | 20.69 | 0.32 | 0.83 | 1.32 |
| (S)-(+)-2-Amino-1-propanol | 75.0685 | 22.41 | 20.36 | 21.23 | 21.41 | 21.77 | 21.70 | 0.32 | 0.80 | 1.34 |
| Cotinine | 176.0951 | 2.83 | 19.80 | 19.68 | 20.50 | 20.07 | 21.21 | 0.32 | 0.67 | 1.41 |
| 3-Methylxanthine | 166.0492 | 5.26 | 22.91 | 23.14 | 24.79 | 24.05 | 24.05 | 0.32 | 0.44 | 1.13 |
| (8aR.12S.12aR)-12-Hydroxy-4-methyl-4.5.6.7.8.8a.12.12a-octahydro-2H-3-benzoxecine-2.9(1H)-dione | 252.1362 | 2.24 | 17.73 | 17.48 | 19.01 | 18.57 | 18.77 | 0.32 | 0.56 | 1.04 |
| 3-Methylglutaric acid | 146.0581 | 14.50 | 17.27 | 16.91 | 18.25 | 18.58 | 18.02 | 0.32 | 0.52 | 0.75 |
| 5-Methylcytosine | 125.0590 | 14.40 | 19.27 | 19.93 | 20.09 | 20.63 | 20.51 | 0.32 | 0.87 | 1.24 |
| 4-Hydroxybutyric acid (GHB) | 104.0475 | 4.10 | 21.93 | 21.83 | 22.85 | 22.69 | 23.09 | 0.32 | 0.79 | 1.15 |
| Isoleucine | 131.0948 | 12.84 | 18.06 | 18.31 | 18.96 | 19.08 | 19.24 | 0.31 | 0.92 | 1.18 |
| 4-Dodecylbenzenesulfonic acid | 326.1918 | 1.76 | 18.12 | 18.04 | 18.39 | 18.67 | 19.35 | 0.31 | 0.85 | 1.23 |
| L-Pyroglutamic acid | 129.0427 | 3.00 | 22.19 | 23.16 | 23.30 | 23.65 | 23.49 | 0.31 | 0.73 | 1.30 |
| Benzylpiperazine | 176.1315 | 18.87 | 19.35 | 19.68 | 19.64 | 20.10 | 20.69 | 0.31 | 0.89 | 1.34 |
| Valylproline | 214.1318 | 7.54 | 22.48 | 22.59 | 23.18 | 23.42 | 23.59 | 0.30 | 0.95 | 1.10 |
| 6-Aminonicotinic acid | 138.0430 | 8.84 | 21.80 | 22.28 | 22.58 | 23.04 | 22.93 | 0.30 | 0.90 | 1.14 |
| 3-Hydroxybutyric acid | 104.0475 | 5.42 | 21.59 | 21.66 | 22.57 | 22.38 | 22.75 | 0.30 | 0.81 | 1.15 |
| L-Hydroxyproline | 131.0584 | 15.28 | 19.62 | 18.86 | 20.89 | 20.00 | 20.55 | 0.30 | 0.35 | 0.93 |
| N-Isovalerylglycine | 159.0897 | 5.33 | 19.81 | 20.12 | 20.79 | 20.80 | 20.96 | 0.30 | 0.88 | 1.14 |
| Creatine | 131.0695 | 15.50 | 26.98 | 27.00 | 27.28 | 27.02 | 28.43 | 0.29 | 0.56 | 1.45 |
| 2-Hydroxyhippuric acid | 195.0532 | 7.70 | 23.72 | 24.41 | 24.55 | 24.71 | 25.02 | 0.29 | 0.90 | 1.30 |
| 3-Methylsalicylic acid | 152.0475 | 7.40 | 24.67 | 25.82 | 26.47 | 26.17 | 25.92 | 0.28 | 0.43 | 1.24 |
| 4-Methylphenol | 108.0576 | 7.40 | 22.84 | 23.96 | 24.58 | 24.31 | 24.08 | 0.28 | 0.45 | 1.25 |
| N-α-L-Acetyl-arginine | 216.1223 | 15.79 | 23.34 | 23.74 | 24.06 | 24.45 | 24.41 | 0.28 | 0.92 | 1.06 |
| α-Lactose | 359.1426 | 16.24 | 20.09 | 20.25 | 21.13 | 20.89 | 21.18 | 0.28 | 0.77 | 1.09 |
| 2-Furoic acid | 112.0161 | 19.88 | 19.46 | 20.25 | 20.30 | 20.62 | 20.67 | 0.28 | 0.83 | 1.21 |
| Guvacoline | 141.0791 | 5.34 | 24.32 | 24.68 | 25.30 | 25.25 | 25.42 | 0.28 | 0.86 | 1.10 |
| Betaine | 117.0791 | 10.27 | 24.59 | 24.62 | 25.07 | 25.22 | 25.66 | 0.27 | 0.94 | 1.07 |
| L-(+)-Citrulline | 175.0958 | 16.44 | 21.62 | 21.80 | 22.44 | 22.53 | 22.62 | 0.27 | 0.89 | 1.00 |
| DL-Stachydrine | 143.0947 | 13.52 | 23.73 | 24.12 | 24.64 | 24.71 | 24.80 | 0.27 | 0.89 | 1.07 |
| 7-Methyladenine | 149.0703 | 15.94 | 20.93 | 21.49 | 21.59 | 22.07 | 21.98 | 0.27 | 0.87 | 1.05 |
| N-Phenylacetylglutamine | 264.1109 | 3.00 | 27.46 | 28.51 | 28.65 | 28.87 | 28.62 | 0.27 | 0.58 | 1.16 |
| (-)-Camphanic acid | 198.0893 | 3.08 | 20.28 | 18.57 | 20.11 | 20.78 | 20.51 | 0.27 | 0.24 | 0.23 |
| L-Pyroglutamic acid | 129.0427 | 4.56 | 22.40 | 23.24 | 23.39 | 23.66 | 23.51 | 0.26 | 0.71 | 1.11 |
| 2.2-Bis(hydroxymethyl)propionic acid | 134.0580 | 6.75 | 20.93 | 22.68 | 22.60 | 22.10 | 22.52 | 0.26 | 0.32 | 1.58 |
| L-Histidine | 155.0696 | 15.35 | 20.24 | 20.75 | 21.23 | 21.35 | 21.23 | 0.26 | 0.78 | 0.99 |
| Uracil | 112.0274 | 5.92 | 20.00 | 20.46 | 20.64 | 21.24 | 20.89 | 0.26 | 0.76 | 0.89 |
| Levulinic acid | 116.0475 | 2.84 | 21.55 | 21.59 | 22.48 | 22.09 | 22.57 | 0.25 | 0.71 | 1.02 |
| 1.3.7-Trimethyluric acid | 210.0755 | 2.63 | 19.85 | 21.48 | 20.46 | 21.06 | 21.33 | 0.25 | 0.36 | 1.48 |
| Taurine | 125.0147 | 15.01 | 19.51 | 20.02 | 20.10 | 20.33 | 20.62 | 0.25 | 0.94 | 1.11 |
| 1.3-Dimethyluric acid | 196.0597 | 4.65 | 22.84 | 24.23 | 23.60 | 24.13 | 24.14 | 0.25 | 0.46 | 1.31 |
| Crotonic acid | 86.0368 | 20.83 | 19.00 | 19.37 | 19.76 | 19.73 | 20.08 | 0.25 | 0.92 | 1.08 |
| sn-Glycerol-3-phosphate | 172.0139 | 16.31 | 18.77 | 19.16 | 19.48 | 19.82 | 19.69 | 0.25 | 0.87 | 0.92 |
| L-(-)-Asparagine | 132.0536 | 16.23 | 19.04 | 19.29 | 19.77 | 19.74 | 20.07 | 0.25 | 0.93 | 1.03 |
| 4-Hydroxybutyric acid (GHB) | 104.0475 | 3.00 | 21.72 | 21.63 | 22.60 | 22.12 | 22.72 | 0.25 | 0.63 | 1.00 |
| L-(+)-Alanine | 89.0478 | 15.48 | 21.62 | 21.76 | 22.50 | 22.36 | 22.55 | 0.25 | 0.80 | 0.93 |
| Benzoic acid | 122.0369 | 7.40 | 21.37 | 22.48 | 23.18 | 22.85 | 22.41 | 0.25 | 0.33 | 1.05 |
| 3-Indoxyl sulphate | 213.0097 | 2.07 | 19.28 | 18.16 | 18.68 | 18.41 | 20.38 | 0.25 | 0.19 | 1.10 |
| 3-Methylhistamine | 125.0954 | 23.23 | 18.78 | 19.24 | 19.82 | 19.83 | 19.70 | 0.24 | 0.70 | 0.92 |
| Xanthine | 152.0335 | 10.29 | 20.11 | 20.57 | 20.42 | 21.22 | 21.00 | 0.24 | 0.74 | 0.88 |
| 2-Furoic acid | 112.0162 | 17.15 | 17.83 | 18.13 | 17.80 | 17.80 | 19.20 | 0.24 | 0.40 | 1.36 |
| Argininosuccinic acid | 290.1225 | 18.42 | 16.73 | 17.12 | 17.38 | 17.68 | 17.64 | 0.24 | 0.91 | 0.91 |
| 1.3-Dimethyluric acid | 196.0598 | 3.00 | 21.66 | 23.13 | 22.50 | 22.90 | 22.96 | 0.24 | 0.41 | 1.30 |
| Pipecolic acid | 129.0791 | 12.34 | 18.58 | 18.67 | 19.51 | 19.23 | 19.48 | 0.24 | 0.71 | 0.90 |
| L-(-)-Phenylalanine | 165.0791 | 9.87 | 22.39 | 22.86 | 22.95 | 22.90 | 23.53 | 0.23 | 0.82 | 1.14 |
| Threonine | 119.0583 | 8.52 | 20.79 | 21.29 | 21.94 | 21.54 | 21.82 | 0.23 | 0.63 | 1.04 |
| Stearic acid | 284.2716 | 1.85 | 21.49 | 21.19 | 21.46 | 21.44 | 22.52 | 0.23 | 0.50 | 1.03 |
| 3-Hydroxy-3-methylglutaric acid | 162.0530 | 14.49 | 22.10 | 22.59 | 22.54 | 23.06 | 23.02 | 0.23 | 0.86 | 0.91 |
| L-(-)-Methionine | 149.0511 | 11.37 | 18.36 | 18.77 | 18.91 | 18.63 | 19.57 | 0.23 | 0.64 | 1.21 |
| cis-Aconitic acid | 174.0165 | 18.66 | 24.93 | 25.38 | 25.51 | 25.83 | 25.85 | 0.23 | 0.92 | 0.92 |
| L-(-)-Serine | 105.0426 | 16.77 | 19.56 | 19.88 | 20.39 | 20.30 | 20.50 | 0.23 | 0.86 | 0.93 |
| 2-Hydroxyhippuric acid | 195.0533 | 8.16 | 23.67 | 24.32 | 23.91 | 24.83 | 24.56 | 0.23 | 0.59 | 0.89 |
| 4-Coumaric acid | 164.0475 | 13.46 | 18.32 | 19.02 | 18.91 | 19.05 | 19.45 | 0.23 | 0.79 | 1.13 |
| Pipecolic acid | 129.0791 | 10.00 | 19.83 | 18.91 | 20.61 | 19.91 | 20.46 | 0.23 | 0.28 | 0.63 |
| N-Phenylacetylglutamine | 264.1109 | 4.56 | 28.04 | 28.81 | 28.88 | 29.23 | 28.96 | 0.22 | 0.64 | 0.91 |
| Cytisine | 190.1107 | 20.02 | 18.10 | 18.50 | 18.28 | 18.90 | 19.02 | 0.22 | 0.81 | 0.92 |
| Prolinamide | 114.0794 | 8.98 | 21.03 | 21.28 | 21.80 | 21.83 | 21.87 | 0.22 | 0.85 | 0.84 |
| Adenosine | 267.0966 | 7.77 | 18.80 | 19.46 | 19.86 | 19.89 | 19.69 | 0.22 | 0.61 | 0.89 |
| Citraconic acid | 130.0268 | 18.66 | 23.59 | 23.93 | 24.07 | 24.44 | 24.45 | 0.22 | 0.94 | 0.85 |
| Crotonic acid | 86.0368 | 18.66 | 20.92 | 21.30 | 21.46 | 21.77 | 21.79 | 0.22 | 0.93 | 0.87 |
| L-threo-3-Phenylserine | 181.0740 | 4.63 | 19.60 | 19.58 | 19.48 | 19.79 | 20.59 | 0.22 | 0.59 | 0.99 |
| Citric acid | 192.0272 | 20.60 | 24.70 | 25.17 | 25.14 | 25.60 | 25.57 | 0.22 | 0.86 | 0.87 |
| D-(+)-Malic acid | 134.0217 | 17.43 | 23.71 | 23.70 | 23.96 | 24.11 | 24.58 | 0.21 | 0.88 | 0.87 |
| Pipecolic acid | 129.0791 | 23.50 | 18.61 | 18.79 | 19.23 | 19.16 | 19.47 | 0.21 | 0.90 | 0.86 |
| 2-Furoic acid | 112.0161 | 18.66 | 20.05 | 20.31 | 20.47 | 20.86 | 20.82 | 0.21 | 0.92 | 0.77 |
| Methylimidazoleacetic acid | 140.0586 | 6.68 | 26.93 | 27.04 | 27.30 | 27.59 | 27.70 | 0.21 | 0.97 | 0.76 |
| Cyclamic acid | 179.0618 | 2.47 | 26.63 | 27.37 | 27.42 | 27.86 | 27.42 | 0.21 | 0.54 | 0.79 |
| L-Cystine | 240.0239 | 17.24 | 19.49 | 18.96 | 19.56 | 19.40 | 20.30 | 0.20 | 0.45 | 0.80 |
| 7-Methylxanthine | 166.0492 | 5.70 | 22.93 | 23.81 | 23.71 | 23.79 | 23.96 | 0.20 | 0.63 | 1.02 |
| L-(+)-Tartaric acid | 150.0165 | 18.45 | 22.08 | 22.70 | 22.04 | 23.18 | 22.86 | 0.20 | 0.41 | 0.78 |
| DL-Tryptophan | 204.0899 | 11.77 | 21.85 | 22.19 | 22.36 | 22.39 | 22.76 | 0.20 | 0.93 | 0.91 |
| N-Acetylalanine | 131.0583 | 15.85 | 17.06 | 17.42 | 17.65 | 17.76 | 17.90 | 0.20 | 0.94 | 0.84 |
| Meso-erythritol | 122.0580 | 10.41 | 22.66 | 22.98 | 23.24 | 23.41 | 23.45 | 0.20 | 0.93 | 0.78 |
| N6.N6.N6-Trimethyl-L-lysine | 188.1525 | 23.49 | 24.89 | 24.96 | 25.57 | 25.38 | 25.68 | 0.20 | 0.79 | 0.79 |
| 5-Methylcytosine | 125.0590 | 10.54 | 20.46 | 21.24 | 20.55 | 21.53 | 21.31 | 0.20 | 0.43 | 0.85 |
| Guanidineacetic acid | 117.0539 | 16.91 | 22.55 | 22.80 | 23.11 | 23.18 | 23.35 | 0.20 | 0.96 | 0.80 |
| trans-Aconitic acid | 174.0165 | 16.25 | 21.50 | 21.65 | 21.98 | 22.17 | 22.20 | 0.19 | 0.94 | 0.70 |
| Methylsuccinic acid | 132.0423 | 12.65 | 17.93 | 18.24 | 18.32 | 18.68 | 18.66 | 0.19 | 0.92 | 0.73 |
| 4-Acetamidobutanoic acid | 145.0740 | 10.95 | 21.77 | 21.60 | 22.60 | 21.83 | 22.61 | 0.19 | 0.39 | 0.84 |
| N4-Acetylcytidine | 285.0960 | 6.88 | 22.07 | 22.30 | 22.72 | 22.72 | 22.81 | 0.19 | 0.87 | 0.74 |
| Suberic acid | 174.0893 | 11.47 | 18.53 | 18.96 | 19.44 | 19.33 | 19.29 | 0.19 | 0.65 | 0.76 |
| Citric acid | 192.0272 | 19.88 | 26.25 | 26.66 | 26.76 | 27.09 | 26.98 | 0.19 | 0.84 | 0.73 |
| Acetyl-β-methylcholine | 159.1260 | 13.33 | 25.99 | 26.02 | 26.69 | 26.48 | 26.69 | 0.19 | 0.71 | 0.70 |
| N-Acetylaspartic acid | 175.0482 | 15.85 | 22.01 | 22.19 | 22.36 | 22.62 | 22.73 | 0.19 | 0.99 | 0.72 |
| 3-Methyladipic acid | 160.0737 | 13.18 | 20.03 | 20.39 | 20.76 | 20.81 | 20.75 | 0.19 | 0.77 | 0.72 |
| L-Carnitine | 161.1053 | 13.10 | 17.75 | 17.57 | 18.39 | 18.19 | 18.36 | 0.18 | 0.61 | 0.60 |
| 1-Aminocyclohexanecarboxylic acid | 143.0947 | 9.46 | 27.16 | 27.04 | 27.65 | 27.52 | 27.84 | 0.18 | 0.76 | 0.68 |
| Bis(4-ethylbenzylidene)sorbitol | 414.2041 | 2.19 | 20.00 | 20.11 | 20.75 | 21.02 | 20.46 | 0.18 | 0.45 | 0.45 |
| Valylproline | 214.1318 | 3.09 | 21.29 | 21.46 | 21.70 | 21.87 | 21.97 | 0.18 | 0.98 | 0.68 |
| N-(1-Benzylpiperidin-4-yl)-6-phenylthieno[3.2-d]pyrimidin-4-amine | 400.1736 | 2.77 | 20.70 | 21.19 | 22.41 | 21.39 | 21.49 | 0.18 | 0.20 | 0.79 |
| Propionylcarnitine | 217.1316 | 10.79 | 18.57 | 18.91 | 19.41 | 19.10 | 19.36 | 0.18 | 0.66 | 0.79 |
| L-(-)-Tyrosine | 181.0741 | 13.46 | 21.22 | 22.14 | 21.73 | 21.87 | 22.23 | 0.18 | 0.48 | 1.01 |
| L-2-Aminoadipic acid | 161.0689 | 16.03 | 18.57 | 18.58 | 19.31 | 18.91 | 19.26 | 0.17 | 0.58 | 0.69 |
| L-(+)-Arginine | 174.1118 | 26.50 | 20.58 | 20.78 | 21.05 | 20.76 | 21.44 | 0.17 | 0.65 | 0.86 |
| Choline | 103.0997 | 22.21 | 24.04 | 24.23 | 24.28 | 24.41 | 24.81 | 0.17 | 0.89 | 0.76 |
| Hippuric acid | 179.0584 | 4.03 | 25.72 | 26.06 | 26.25 | 26.52 | 26.34 | 0.17 | 0.78 | 0.61 |
| Ethylmalonic acid | 132.0424 | 11.69 | 19.48 | 19.76 | 19.94 | 20.29 | 20.05 | 0.17 | 0.75 | 0.57 |
| Monodesmethylisoproturon | 192.1264 | 23.27 | 19.52 | 19.74 | 19.60 | 19.93 | 20.25 | 0.17 | 0.80 | 0.74 |
| 3-Aminosalicylic acid | 153.0426 | 4.60 | 21.16 | 21.57 | 21.94 | 21.88 | 21.83 | 0.17 | 0.66 | 0.67 |
| Orotic acid | 156.0173 | 7.02 | 19.65 | 19.88 | 19.89 | 20.18 | 20.32 | 0.16 | 0.95 | 0.68 |
| S-Adenosyl-L-homocysteine | 384.1214 | 14.74 | 17.63 | 17.90 | 18.10 | 18.22 | 18.27 | 0.16 | 0.94 | 0.64 |
| L-(+)-Glutamine | 146.0692 | 16.12 | 24.50 | 24.69 | 24.97 | 25.18 | 25.05 | 0.16 | 0.82 | 0.55 |
| L-Kynurenine | 208.0849 | 11.00 | 20.29 | 20.16 | 20.39 | 20.38 | 20.98 | 0.16 | 0.64 | 0.68 |
| Methylimidazoleacetic acid | 140.0586 | 3.08 | 25.21 | 25.54 | 25.77 | 25.68 | 25.93 | 0.16 | 0.83 | 0.72 |
| 3.4-Dihydroxybenzenesulfonic acid | 189.9938 | 4.65 | 22.27 | 21.97 | 23.71 | 22.34 | 22.86 | 0.15 | 0.13 | 0.58 |
| N-(1-Benzylpiperidin-4-yl)-6-phenylthieno[3.2-d]pyrimidin-4-amine | 400.1735 | 3.60 | 19.29 | 19.77 | 20.68 | 20.06 | 19.92 | 0.15 | 0.23 | 0.63 |
| Sucrose | 342.1164 | 16.24 | 21.74 | 21.86 | 22.59 | 22.02 | 22.42 | 0.15 | 0.43 | 0.68 |
| D-(+)-Arabitol | 152.0686 | 12.16 | 22.68 | 22.98 | 23.25 | 23.23 | 23.31 | 0.15 | 0.84 | 0.63 |
| N-Acetyl-L-tyrosine | 223.0846 | 4.90 | 19.85 | 20.00 | 20.19 | 20.37 | 20.40 | 0.15 | 0.96 | 0.55 |
| L-Ascorbic acid 2-sulfate | 255.9891 | 22.23 | 23.58 | 23.80 | 24.10 | 24.05 | 24.16 | 0.14 | 0.85 | 0.59 |
| 1-Aminocyclohexanecarboxylic acid | 143.0947 | 9.98 | 27.42 | 27.65 | 27.97 | 27.94 | 27.99 | 0.14 | 0.81 | 0.57 |
| N3.N4-Dimethyl-L-arginine | 202.1430 | 23.14 | 26.39 | 26.57 | 26.88 | 26.86 | 26.94 | 0.14 | 0.86 | 0.55 |
| Propionylcarnitine | 217.1314 | 9.20 | 24.14 | 23.89 | 24.90 | 24.23 | 24.66 | 0.14 | 0.29 | 0.52 |
| Urocanic acid | 138.0430 | 10.70 | 21.77 | 22.52 | 21.55 | 22.79 | 22.32 | 0.14 | 0.18 | 0.55 |
| Hippuric acid | 179.0584 | 2.94 | 25.89 | 26.11 | 26.45 | 26.46 | 26.39 | 0.13 | 0.73 | 0.50 |
| Trigonelline | 137.0478 | 11.07 | 25.95 | 26.18 | 26.89 | 26.31 | 26.56 | 0.13 | 0.35 | 0.61 |
| Theophylline | 180.0648 | 4.19 | 18.99 | 19.59 | 19.17 | 19.12 | 19.87 | 0.13 | 0.31 | 0.88 |
| Kynurenic acid | 189.0427 | 2.99 | 20.20 | 20.46 | 20.25 | 20.57 | 20.80 | 0.13 | 0.72 | 0.59 |
| N-Isovalerylglycine | 159.0897 | 2.65 | 20.45 | 20.86 | 21.09 | 21.05 | 20.98 | 0.12 | 0.58 | 0.53 |
| 9-Methyluric acid | 182.0442 | 8.05 | 25.10 | 25.86 | 26.36 | 25.65 | 25.79 | 0.12 | 0.17 | 0.69 |
| Hydroxylysine | 162.1005 | 24.71 | 16.88 | 16.73 | 17.09 | 16.83 | 17.41 | 0.12 | 0.46 | 0.53 |
| 3-Methylhistidine | 169.0852 | 12.90 | 24.77 | 24.50 | 25.42 | 25.04 | 25.07 | 0.11 | 0.27 | 0.30 |
| 1-(Carboxymethyl)cyclohexanecarboxylic acid | 186.0893 | 11.35 | 19.37 | 19.50 | 19.65 | 19.63 | 19.88 | 0.11 | 0.92 | 0.51 |
| N-Acetyl-L-carnosine | 268.1171 | 3.05 | 20.71 | 21.13 | 21.11 | 21.09 | 21.29 | 0.11 | 0.69 | 0.58 |
| L-Proline | 115.0634 | 12.47 | 22.38 | 22.29 | 22.68 | 22.27 | 22.93 | 0.11 | 0.36 | 0.56 |
| Indole-3-lactic acid | 205.0740 | 4.63 | 21.48 | 22.24 | 21.86 | 22.17 | 22.06 | 0.11 | 0.32 | 0.58 |
| 6-Aminocaproic acid | 131.0947 | 23.09 | 18.07 | 18.47 | 18.34 | 18.51 | 18.58 | 0.11 | 0.69 | 0.51 |
| Imidazolelactic acid | 156.0536 | 5.80 | 19.96 | 20.08 | 20.30 | 20.37 | 20.34 | 0.10 | 0.85 | 0.38 |
| Fumaric acid | 116.0110 | 17.48 | 19.77 | 19.71 | 20.09 | 18.97 | 20.66 | 0.10 | 0.07 | 0.89 |
| L-Leucine | 131.0947 | 10.53 | 23.40 | 23.88 | 23.54 | 23.84 | 23.94 | 0.10 | 0.49 | 0.54 |
| Citraconic acid | 130.0268 | 20.85 | 22.17 | 22.49 | 22.51 | 22.52 | 22.67 | 0.10 | 0.79 | 0.49 |
| Tyrosol | 138.0682 | 3.00 | 17.72 | 18.11 | 18.18 | 17.96 | 18.28 | 0.10 | 0.50 | 0.56 |
| Pseudouridine | 244.0696 | 11.73 | 25.21 | 25.52 | 25.66 | 25.65 | 25.63 | 0.10 | 0.66 | 0.42 |
| Hexanoylcarnitine | 259.1784 | 5.83 | 20.90 | 20.99 | 21.18 | 21.23 | 21.26 | 0.10 | 0.92 | 0.36 |
| Isophthalic acid | 166.0267 | 17.31 | 18.17 | 17.53 | 17.22 | 17.14 | 18.84 | 0.10 | 0.04 | 0.67 |
| 3-Ureidopropionic acid | 132.0536 | 11.33 | 21.13 | 21.18 | 20.55 | 21.72 | 21.32 | 0.09 | 0.12 | 0.19 |
| N-Tigloylglycine | 157.0741 | 2.80 | 21.89 | 22.42 | 22.50 | 22.37 | 22.36 | 0.09 | 0.35 | 0.47 |
| Imidazoleacetic acid | 126.0430 | 13.73 | 18.44 | 18.83 | 18.67 | 18.94 | 18.82 | 0.09 | 0.51 | 0.38 |
| Kynurenic acid | 189.0427 | 4.35 | 22.50 | 22.74 | 22.29 | 22.83 | 22.86 | 0.08 | 0.29 | 0.36 |
| α-Lactose | 342.1163 | 17.30 | 19.33 | 19.37 | 19.71 | 19.66 | 19.59 | 0.08 | 0.55 | 0.26 |
| 4-Chlorophenoxyacetic acid | 186.0085 | 2.26 | 19.19 | 19.51 | 20.05 | 19.14 | 19.78 | 0.08 | 0.11 | 0.59 |
| (-)-Camphanic acid | 198.0893 | 10.45 | 19.81 | 20.08 | 19.90 | 20.17 | 20.16 | 0.08 | 0.60 | 0.35 |
| Acetylcholine | 145.1103 | 13.35 | 23.23 | 23.02 | 23.78 | 23.29 | 23.46 | 0.07 | 0.16 | 0.23 |
| 3-Hydroxy-3-methylglutaric acid | 162.0530 | 16.23 | 22.00 | 22.40 | 22.09 | 22.49 | 22.31 | 0.07 | 0.30 | 0.32 |
| 4-Acetamidobutanoic acid | 145.0739 | 4.74 | 21.71 | 21.53 | 22.21 | 21.83 | 21.92 | 0.07 | 0.20 | 0.20 |
| L-Glutamic acid | 147.0533 | 18.98 | 20.64 | 20.87 | 20.71 | 21.18 | 20.84 | 0.07 | 0.29 | 0.20 |
| 3-tert-Butyladipic acid | 202.1207 | 2.97 | 21.12 | 21.38 | 21.77 | 21.42 | 21.45 | 0.07 | 0.23 | 0.33 |
| 4-Oxoproline | 129.0428 | 7.49 | 26.37 | 26.45 | 26.83 | 26.54 | 26.68 | 0.07 | 0.37 | 0.30 |
| Glycine | 75.0321 | 16.72 | 20.10 | 19.66 | 19.63 | 19.52 | 20.50 | 0.07 | 0.06 | 0.40 |
| Urea | 60.0327 | 8.81 | 25.99 | 26.11 | 26.30 | 26.21 | 26.28 | 0.07 | 0.68 | 0.28 |
| Acetyl-L-carnitine | 203.1158 | 10.40 | 29.34 | 29.21 | 29.62 | 29.37 | 29.57 | 0.06 | 0.34 | 0.23 |
| N-Acetylneuraminic acid | 309.1060 | 13.07 | 24.58 | 24.83 | 24.80 | 24.86 | 24.86 | 0.06 | 0.63 | 0.27 |
| Choline glycerophosphate | 257.1029 | 15.24 | 21.50 | 21.54 | 21.69 | 21.90 | 21.60 | 0.06 | 0.32 | 0.11 |
| S-(5'-Adenosyl)-L-methionine (SAM) | 398.1373 | 18.66 | 18.19 | 18.33 | 18.47 | 18.12 | 18.58 | 0.06 | 0.22 | 0.39 |
| 2-Isopropylmalic acid | 176.0685 | 14.38 | 18.53 | 18.75 | 18.47 | 18.93 | 18.73 | 0.06 | 0.24 | 0.19 |
| 4-Acetamidobutanoic acid | 145.0740 | 3.01 | 23.05 | 23.22 | 22.87 | 23.05 | 23.41 | 0.06 | 0.18 | 0.36 |
| L-Creatinine | 113.0590 | 8.08 | 28.49 | 28.68 | 28.66 | 28.69 | 28.71 | 0.05 | 0.65 | 0.22 |
| Gluconic acid | 196.0584 | 12.71 | 25.82 | 26.34 | 26.59 | 26.06 | 26.18 | 0.04 | 0.06 | 0.36 |
| Androsterone glucuronide | 466.2569 | 2.17 | 21.99 | 22.14 | 22.10 | 22.28 | 22.14 | 0.04 | 0.46 | 0.15 |
| L-(-)-Threonine | 119.0584 | 14.89 | 20.82 | 21.04 | 20.90 | 20.98 | 21.07 | 0.04 | 0.45 | 0.25 |
| Esculin | 340.0814 | 12.90 | 20.40 | 20.54 | 20.54 | 20.57 | 20.61 | 0.04 | 0.79 | 0.21 |
| L-(+)-Cystathionine | 222.0674 | 18.49 | 16.93 | 16.39 | 17.09 | 16.39 | 17.14 | 0.04 | 0.03 | 0.21 |
| DL-Methionine sulfoxide | 165.0461 | 13.64 | 18.72 | 18.66 | 18.33 | 18.30 | 19.11 | 0.04 | 0.04 | 0.39 |
| L-(+)-Valine | 117.0791 | 12.17 | 18.28 | 18.42 | 18.34 | 18.16 | 18.61 | 0.04 | 0.14 | 0.33 |
| 3-Butene-1.2.3-tricarboxylic acid | 188.0322 | 15.74 | 20.16 | 20.47 | 20.10 | 20.23 | 20.46 | 0.04 | 0.11 | 0.30 |
| DL-3-Hydroxykyunrenine | 224.0797 | 13.43 | 22.15 | 21.83 | 21.61 | 22.09 | 22.20 | 0.04 | 0.05 | 0.05 |
| Suberic acid | 174.0893 | 7.66 | 19.78 | 20.20 | 19.94 | 19.94 | 20.06 | 0.03 | 0.10 | 0.28 |
| Uric Acid | 168.0284 | 11.95 | 27.08 | 27.08 | 27.20 | 27.07 | 27.24 | 0.03 | 0.38 | 0.16 |
| N-Acetyl-1-aspartylglutamic acid | 304.0907 | 19.13 | 18.78 | 18.95 | 18.87 | 19.01 | 18.89 | 0.03 | 0.26 | 0.11 |
| Saccharin | 182.9992 | 2.50 | 26.10 | 26.29 | 25.44 | 26.95 | 25.91 | 0.03 | 0.01 | -0.19 |
| L-Aspartic acid | 133.0376 | 19.39 | 17.86 | 17.95 | 17.92 | 17.98 | 17.98 | 0.03 | 0.73 | 0.12 |
| Imidazoleacetic acid | 126.0430 | 12.83 | 20.59 | 20.91 | 20.74 | 20.90 | 20.72 | 0.03 | 0.09 | 0.13 |
| Uridine | 244.0697 | 8.97 | 16.49 | 16.95 | 16.61 | 16.85 | 16.66 | 0.02 | 0.04 | 0.17 |
| 4-Acetamidobutanoic acid | 145.0740 | 5.25 | 24.25 | 24.48 | 24.40 | 24.49 | 24.35 | 0.02 | 0.12 | 0.10 |
| 4-Aminohippuric acid | 194.0692 | 4.60 | 21.94 | 22.24 | 21.88 | 22.39 | 21.92 | 0.01 | 0.01 | -0.02 |
| 2-Oxindole | 133.0528 | 4.29 | 24.74 | 24.84 | 24.95 | 24.88 | 24.75 | 0.01 | 0.01 | 0.01 |
| D-(-)-Ribose | 150.0529 | 11.70 | 24.79 | 24.87 | 24.98 | 24.74 | 24.89 | 0.01 | 0.01 | 0.10 |
| 2-Isopropylmalic acid | 176.0686 | 4.05 | 21.23 | 20.24 | 19.47 | 20.15 | 21.21 | -0.01 | 0.00 | -0.02 |
| Orotidine | 288.0595 | 11.44 | 21.25 | 21.22 | 21.17 | 21.20 | 21.20 | -0.01 | 0.45 | -0.05 |
| α-Hydroxyhippuric acid | 195.0533 | 14.20 | 18.69 | 18.91 | 18.18 | 18.74 | 18.71 | -0.01 | 0.01 | 0.02 |
| L-(+)-Lysine | 146.1056 | 25.78 | 20.90 | 20.73 | 21.05 | 20.49 | 20.95 | -0.01 | 0.01 | 0.05 |
| L-Ornithine | 132.0899 | 24.54 | 18.29 | 18.13 | 18.39 | 17.84 | 18.37 | -0.01 | 0.01 | 0.08 |
| Mevalonic acid | 148.0737 | 2.98 | 22.18 | 22.28 | 22.41 | 22.20 | 22.14 | -0.02 | 0.06 | -0.04 |
| δ-Gluconic acid δ-lactone | 178.0479 | 16.60 | 20.11 | 20.18 | 19.92 | 20.20 | 19.98 | -0.02 | 0.09 | -0.13 |
| DL-Malic acid | 134.0217 | 16.72 | 22.50 | 22.75 | 22.56 | 22.62 | 22.44 | -0.03 | 0.11 | -0.06 |
| Furosemide | 330.0080 | 2.21 | 23.79 | 24.14 | 22.35 | 24.33 | 23.57 | -0.03 | 0.00 | -0.22 |
| 4-Pyridoxic acid | 183.0533 | 2.17 | 24.18 | 25.69 | 24.95 | 24.78 | 24.50 | -0.03 | 0.01 | 0.33 |
| 6-Methylnicotinamide | 136.0637 | 25.48 | 26.10 | 26.33 | 26.09 | 26.30 | 25.96 | -0.03 | 0.09 | -0.14 |
| D-Saccharic acid | 210.0377 | 18.93 | 20.34 | 20.85 | 19.98 | 20.82 | 20.19 | -0.03 | 0.02 | -0.15 |
| 3-Butene-1.2.3-tricarboxylic acid | 188.0322 | 18.23 | 19.73 | 19.87 | 19.62 | 19.63 | 19.62 | -0.05 | 0.44 | -0.10 |
| Riboflavin | 376.1372 | 16.04 | 18.15 | 18.20 | 19.15 | 18.21 | 17.81 | -0.07 | 0.05 | -0.34 |
| Metoclopramide | 299.1400 | 7.02 | 23.74 | 24.79 | 25.42 | 24.06 | 23.75 | -0.07 | 0.02 | 0.01 |
| 5-Aminovaleric acid | 117.0791 | 6.09 | 21.39 | 21.52 | 21.12 | 21.16 | 21.21 | -0.07 | 0.46 | -0.18 |
| Trigonelline | 137.0478 | 8.37 | 18.75 | 18.86 | 18.71 | 18.60 | 18.48 | -0.08 | 0.74 | -0.27 |
| Guanidinosuccinic acid | 175.0594 | 16.72 | 26.32 | 26.38 | 26.07 | 26.05 | 25.97 | -0.10 | 0.81 | -0.34 |
| Quinolinic acid | 167.0220 | 16.48 | 22.53 | 22.45 | 22.05 | 22.20 | 22.15 | -0.10 | 0.61 | -0.38 |
| L-Saccharopine | 276.1321 | 17.01 | 19.56 | 19.53 | 19.19 | 19.23 | 19.21 | -0.10 | 0.74 | -0.36 |
| Picolinic acid | 123.0322 | 16.48 | 20.77 | 20.71 | 20.30 | 20.49 | 20.36 | -0.10 | 0.63 | -0.41 |
| D-α-Hydroxyglutaric acid | 148.0373 | 20.93 | 23.78 | 23.88 | 23.51 | 23.78 | 23.30 | -0.10 | 0.49 | -0.47 |
| D-Saccharic acid | 210.0377 | 18.37 | 23.16 | 23.50 | 22.78 | 23.18 | 22.75 | -0.12 | 0.33 | -0.41 |
| L-Norleucine | 131.0947 | 8.44 | 20.86 | 20.71 | 20.78 | 20.39 | 20.43 | -0.12 | 0.78 | -0.43 |
| β-D-Glucopyranuronic acid | 194.0427 | 16.70 | 24.63 | 24.47 | 24.13 | 24.05 | 24.18 | -0.13 | 0.72 | -0.45 |
| 4-Aminoantipyrine | 203.1060 | 2.88 | 20.87 | 21.16 | 20.83 | 20.13 | 20.27 | -0.22 | 0.66 | -0.60 |
| 4-Oxoproline | 129.0428 | 10.81 | 21.09 | 21.16 | 20.21 | 20.72 | 20.06 | -0.25 | 0.62 | -1.03 |
| 3-Hydroxypyridine | 95.0371 | 7.47 | 26.36 | 26.35 | 25.97 | 25.56 | 25.48 | -0.25 | 0.93 | -0.88 |
| 4-Acetamidoantipyrine | 245.1164 | 2.90 | 27.27 | 26.97 | 26.75 | 26.22 | 26.25 | -0.28 | 0.93 | -1.02 |
| Ofloxacin | 361.1437 | 2.92 | 24.54 | 24.37 | 24.08 | 23.43 | 23.50 | -0.30 | 0.91 | -1.04 |
| β-D-Glucopyranuronic acid | 194.0427 | 13.35 | 21.18 | 20.88 | 20.40 | 19.81 | 20.05 | -0.33 | 0.86 | -1.14 |
| 1.5-Anhydro-D-glucitol | 164.0687 | 13.99 | 21.69 | 21.88 | 21.28 | 20.77 | 20.55 | -0.34 | 0.88 | -1.14 |
| Ornithine | 132.0900 | 19.68 | 20.16 | 20.14 | 19.18 | 19.11 | 18.96 | -0.34 | 0.84 | -1.20 |
| D-(-)-Mannitol | 182.0792 | 13.99 | 27.97 | 28.09 | 27.54 | 26.98 | 26.79 | -0.35 | 0.90 | -1.18 |
| N-Formylmethionine | 177.0460 | 6.73 | 22.10 | 22.00 | 20.95 | 21.14 | 20.75 | -0.36 | 0.82 | -1.35 |
| Imidazolelactic acid | 156.0536 | 10.61 | 23.24 | 23.28 | 21.21 | 22.94 | 21.32 | -0.42 | 0.40 | -1.92 |
| 3-tert-Butyladipic acid | 202.1207 | 5.99 | 19.15 | 19.39 | 19.95 | 18.21 | 17.50 | -0.45 | 0.53 | -1.65 |
| 1-(2-Furylmethyl)-5-oxopyrrolidine-3-carboxylic acid | 209.0689 | 2.73 | 20.69 | 20.08 | 19.02 | 18.99 | 18.90 | -0.47 | 0.84 | -1.79 |
| 4-Formylaminoantipyrine | 231.1007 | 2.95 | 20.59 | 19.68 | 19.46 | 18.53 | 18.37 | -0.56 | 0.95 | -2.22 |
| Ofloxacin impurity E | 347.1282 | 2.28 | 19.37 | 19.26 | 17.77 | 17.23 | 17.07 | -0.66 | 0.90 | -2.30 |
| 4-Picoline | 93.0580 | 22.88 | 21.52 | 21.28 | 19.71 | 19.10 | 19.06 | -0.71 | 0.89 | -2.46 |
| Metformin | 129.1016 | 26.71 | 24.72 | 24.52 | 22.75 | 22.37 | 21.90 | -0.78 | 0.92 | -2.82 |
| Mesalamine | 153.0426 | 2.27 | 18.47 | 16.27 | 14.96 | 14.22 | 14.82 | -0.94 | 0.76 | -3.65 |
| Acetaminophen glucuronide | 327.0956 | 11.00 | 18.88 | 18.44 | 17.09 | 16.42 | 15.12 | -0.95 | 0.98 | -3.76 |
| L-Iditol | 182.0792 | 16.90 | 21.48 | 21.15 | 19.26 | 17.81 | 17.41 | -1.15 | 0.95 | -4.07 |
| Moxonidine | 241.0729 | 4.27 | 21.26 | 22.61 | 22.18 | 18.64 | 17.08 | -1.23 | 0.67 | -4.19 |
| Ceftazidime | 546.0995 | 10.82 | 24.54 | 23.76 | 21.58 | 20.31 | 19.92 | -1.27 | 0.95 | -4.62 |
| Acesulfame | 162.9941 | 2.37 | 25.25 | 27.86 | 24.40 | 21.84 | 20.91 | -1.47 | 0.70 | -4.34 |

Dalton (Da), retention time (RT)
